# Development of a microarray based telomerase binding assay reveals unusual binding of a cytochalasin derivative

**DOI:** 10.1038/s41598-025-00230-z

**Published:** 2025-06-04

**Authors:** Jia Li Ye, Lu Fan, Christian Bär, Thomas Thum, Oliver Plettenburg, Russell J. Cox, Carsten Zeilinger

**Affiliations:** 1 Hannover Medical School, Institute of Molecular and Translational Therapeutic Strategies, Carl-Neuberg-Str. 1, 30625 Hannover, Germany; 2 Fraunhofer Institute for Toxicology and Experimental Medicine, Nikolai-Fuchs-Str. 1, 30625 Hannover, Germany; 3 Centre of Biomolecular Drug Research (BMWZ) and Institute for Organic Chemistry, Gottfried-Wilhelm-Leibniz University Hannover, Schneiderberg 38, 30167 Hannover, Germany; 4 Institute of Medicinal Chemistry, Gottfried-Wilhelm-Leibniz University Hannover, Schneiderberg 1B, 30167 Hannover, Germany

**Keywords:** Biotechnology, Cancer, Drug discovery, Biomarkers, Cardiology, Diseases, Medical research

## Abstract

Telomerase reverse transcriptase is crucial for cellular development, regeneration, and disease processes. Strategies for both telomerase activation and inhibition have been intensively explored in the past decades. In this study, we present a highly miniaturized, microarray-based assay designed to identify compounds that target telomerase. The active protein was either recombinantly derived from *E. coli* or obtained from cell lysates of human cancer cell lines and mouse cells expressing telomerase. Using non-contact spotter technology, these lysates or purified telomerase proteins were transferred onto nitrocellulose pads on a microarray. A telomerase binding assay, incorporating fluorescent labelled primer, the template RNA telomerase RNA component, and fluorescent labelled nucleotide as a primer cocktail, was conducted in incubation chambers. Binding of this primer cocktail to spotted telomerase from cell lysates, and from purified recombinant telomerase resulted in an increase in bound fluorescence. Epigallocatechin gallate, a known telomerase inhibitor, reduced this fluorescence in a dose-dependent manner with micromolar affinity. The inhibitory effect on telomerase was validated by thermophoresis and its impact on activity was shown in a Telomerase Repeated Amplification Protocol (TRAP) assay. Additional screening identified that 4’-iodo cytochalasin H inhibits primer cocktail binding to cell lysate in the low micromolar range. Molecular modeling and docking pinpointed a putative binding site for epigallocatechin gallate in a human telomerase homologue, and a putative binding site for 4’-iodo cytochalasin H. In summary, we developed an assay that can be employed to discover new telomerase inhibitors and that will serve as a valuable tool for screening of activators.

## Introduction

Human telomerase is a ribonucleoprotein complex composed of the protein telomerase reverse transcriptase (TERT) and the telomerase RNA component (TERC^1^) that acts as a template. TERT has emerged as a crucial drug target in cancer research due to its reactivation being essential for the unlimited proliferation potential of human cancer cells^2–4^. Numerous TERT-specific compounds designed to inhibit its reverse transcriptase activity have been identified and developed as anti-cancer drugs aimed at limiting tumor growth^5,6^. Conversely, TERT represents a therapeutic target for cell regeneration, as shortened telomeres are associated with various chronic diseases^7^. Moreover, TERT activity is relevant for anti-aging and regenerative therapies since it extends telomeric ends by adding hexameric repeats (TTAGGG)^8,9^. While TERT supplies the enzymatic activity critical for DNA synthesis, TERC serves as the template for forming these telomeric DNA repeats^10^.

The TERT/TERC ribonucleoprotein complex contains two key binding sites: an ATP-binding site and a magnesium-binding site, both of which are essential for its activity^11,12^. The ATP-binding site shares structural homology with the ATP-binding site of HIV reverse transcriptase, which has enabled the repurposing of several antiviral reverse transcriptase inhibitors for cancer treatment^13,14^. However, there are currently no TERT-specific drugs approved by the FDA. The development of TERT inhibitors has become a pivotal focus in anticancer therapy due to their potential to restrict the immortality of cancer cells. Small molecule inhibitors, such as BIBR1532, have been shown to directly interact with TERT, thereby inhibiting its activity^15^. In addition, the natural substance epigallocatechin-3-gallate (EGCG) has shown significant inhibitory effects on TERT function^16^. These inhibitors often function by obstructing the binding sites critical for the enzyme’s function, such as the ATP-binding site and the RNA interaction domain. Targeted approaches have also been explored, such as using antisense oligonucleotides to downregulate TERT expression. For example, GRN163L (Imetelstat), a synthetic oligonucleotide, binds to the RNA template region of TERC, effectively blocking the elongation of telomeres^17^. This inhibition of TERT activity leads to progressive telomere shortening that induces senescence and apoptosis in cancer cells. GRN163L is subject to several past and active clinical trials. One innovative strategy is the use of immunotherapy to target TERT^18^. Vaccines that elicit an immune response against TERT-expressing cells have shown promise in preclinical studies, offering a potential route to specifically target cancer cells while sparing normal cells. Additionally, combination therapies that involve TERT inhibitors and conventional chemotherapy or radiotherapy are being studied to enhance the overall efficacy of cancer treatments^19,20^. These varied approaches underscore the significance of TERT as a multifaceted target in cancer therapy. However, challenges such as the need for improved specificity and reduced off-target effects remain. Continued research and development in this domain hold the potential to yield highly effective therapeutic strategies against cancer and other telomere-related diseases. To facilitate the evaluation of larger compound libraries, simpler assay systems are required in addition to the frequently used but complex telomeric repeat amplification protocol (TRAP) assay^21^. In this study, we investigate the development of an effective nonradioactive chip-based assay for screening potential TERT inhibitors^22–24^. TERT was obtained either as recombinant protein synthesized in *E. coli* and purified via Immobilized Metal Affinity Chromatography (IMAC) or derived from cell culture lysates to test compound binding.

We have developed a microarray-based TERT binding assay that exploits the ability of the TERT complex to bind single-stranded 5’-TTAGGG-3' repeats. It is hypothesized that substances affecting this binding can modulate TERT activity^25,26^. The microarray-based assay revealed that epigallocatechin-3-gallate (EGCG), a known TERT activity inhibitor, demonstrated dose-responsive inhibitory effects. This targeted assay format was employed to test various compounds, including novel cytochalasin derivatives that were generated through mutational and semisynthetic approaches. These derivatives were purified from *Pyricularia grisea* strains and assessed for binding to TERT^27–29^. Cytochalasins are a diverse group of specialised metabolites. Their major cellular target is the polymerisation of actin. However, some non-actin targets are known such as the GLUT1 transporter^30^. Their potential effects on non-actin targets such as TERT may suggest a new avenue for therapeutic intervention^31^.

## Results

**Fig. 1:**
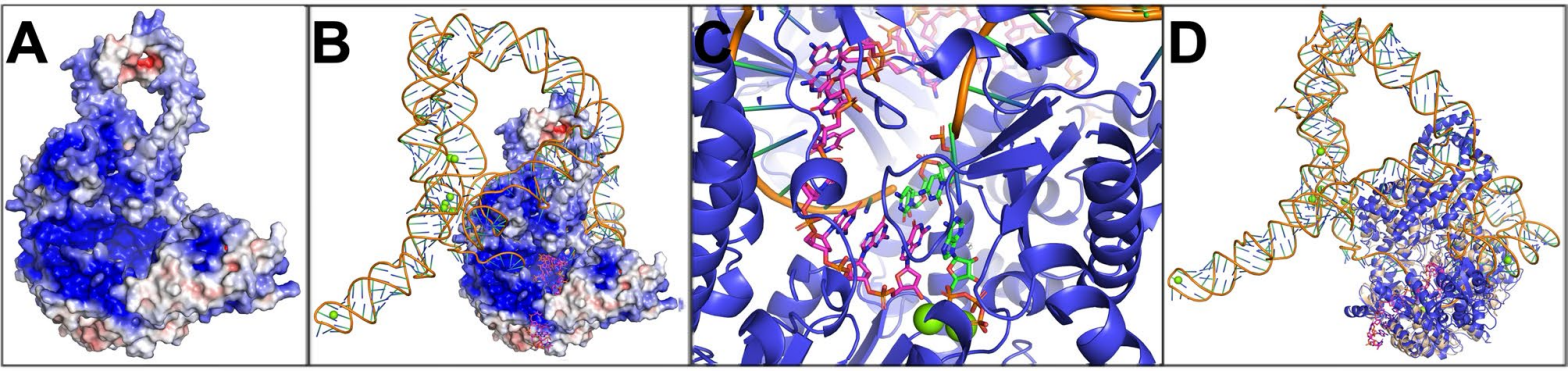
Complex TERT architecture generated by AlphaFold3. (**A**) Visualization of TERT electrostatic surface charges of the protein architecture with a calculated electronegativity between -5 and + 5^21^. (**B**) Presentation of the complex structure of TERT including TERC-RNA sequence in wheat and the nucleotides as tubes whereas the DNA primer given in magenta sticks and magnesium dots in green. (**C**) Inside the TERT cavity at the end of the bound DNA-primer showing three dG´s (magenta sticks), bound ATP with two magnesium ions and complementary paired RNA (green sticks) from TERC sequence. (**D**) Presentation as a cartoon of the alignment of the TERT complex and crystal structure from insect telomerase (pdb: 6USO).

Prior to the development of a binding assay for TERT inhibitors, we investigated the molecular interaction of the known TERT inhibitor, epigallocatechin-3-gallate (EGCG), with TERT through in silico docking experiments. Previous studies have demonstrated the inhibitory activity of EGCG on TERT^25,26,32,33^. The architecture of human TERT, coupled with the RNA sequence TERC, the DNA primer 5’-TTAGGGTTAGGGTTAGGG-3', one ATP molecule, and ten magnesium ions, was modeled using AlphaFold3^34,35^. The modeled TERT structure shows the DNA primer threaded through a pore into a protein cavity, where it is bound in contact with the complementary RNA sequence, while ATP, along with two magnesium ions, is bound at the end of the primer sequence (Fig. 1A–D, TERT1.pdb; Figure S1). Structural alignment with the crystal structure of the insect telomerase from *Tribolium castaneum* (PDB:6USO)—a structural homolog to human TERT—revealed high domain homology (Fig. 1C) providing support for the model structure. The modeled TERT structure without TERC and DNA primer was used for docking experiments with EGCG using the software Diff-Dock-L^36^. The program tested 100 positions for binding at TERT and identified different binding position with a calculated affinity^35–40^. Positions with a binding affinity lower than zero are marked in red (Figure S2A) and mapped to the TERT structure (Figure S2B). Predictions indicate that EGCG could bind with the ATP’s adenine ring or the magnesium ions and phosphate backbone. EGCG predicted a binding affinity of − 3.6 kcal/mol at the nucleotide-binding site (Figure S2C, yellow) and − 2.8 kcal/mol near to a magnesium binding site (white, Figure S2C,^41^).

**Fig. 2:**
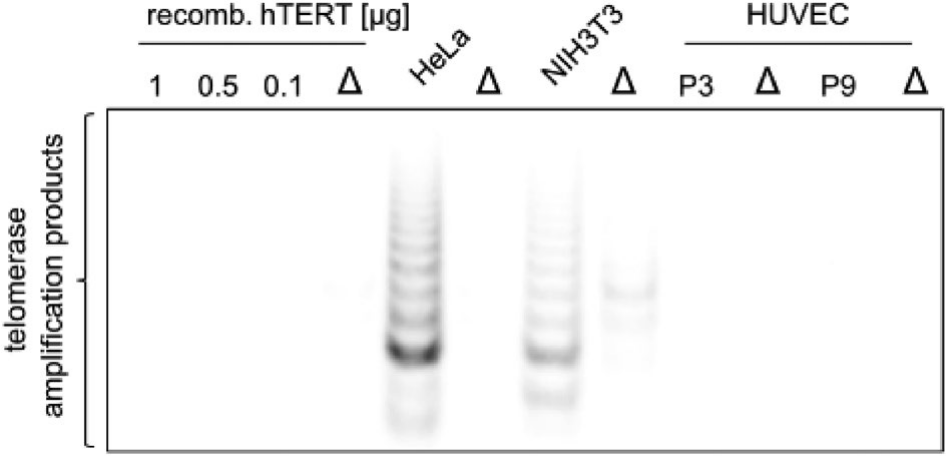
TRAP for detection of telomerase activity. Different concentration of recombinant hTERT isolated from *E. coli* and purified with Ni-IMAC, HeLa, NIH3T3 and HUVEC at passage 3 (P3) and 9 (P9) cell lysates is shown. Δ indicates corresponding heat inactivated cell lysate.

**Fig. 3:**
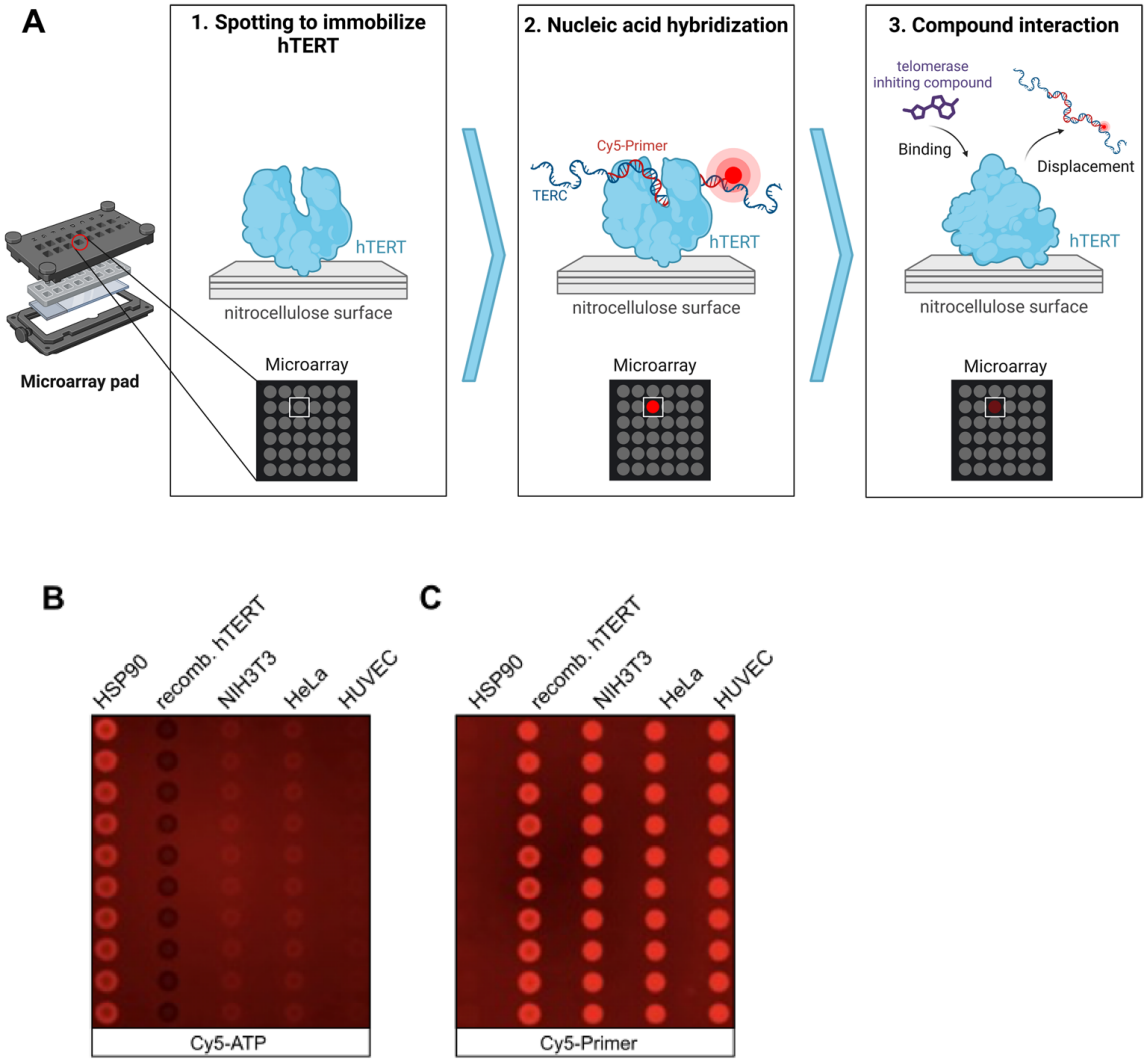
Development of a microarray based binding assay monitoring TERT activity. (**A**) Cell lysates-containing TERT or recombinant expressed become spotted on nitrocellulose pad of a microarray using spotting technology (1). Stabilization of the nucleo-complex at TERT after incubation of microarray slide in chambers with TERC and Cy5-primer (2). Monitoring of bound fluorescence after washing and drying of the microarray slide (3). Lower panel shows the identified fluorescence spots of the microarray. Standard incubation chamber is shown left with inset for separation of pads. (**B**) ATP-binding assay using 100 nM ATP-Cy5 or (**C**) as cocktail containing 10 nM Cy5-primer100 nM dNTP, 0.1 µg TERC-RNA and 10 nM Cy5-ATP diluted in buffer containing 20 mM Hepes, 50 mM KCL, 5 mM MgCl_2_, 0.01% Tween 20, 0.1 mg/mL BSA, 1 mM DTT on spotted proteins (human Hsp90a or recombinant TERT) or cell lysates. The proteins or cell lysates were spotted in columns of ten dots.

**Fig. 4:**
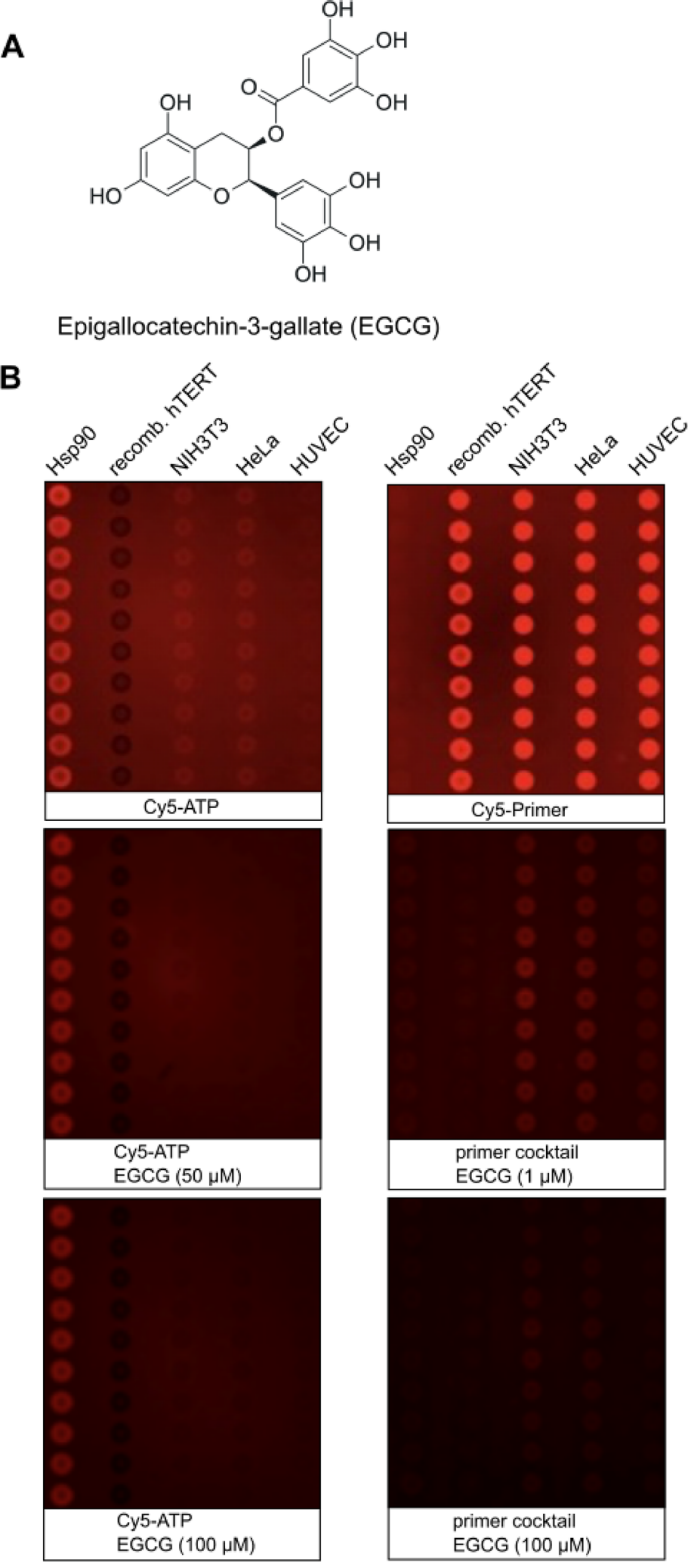

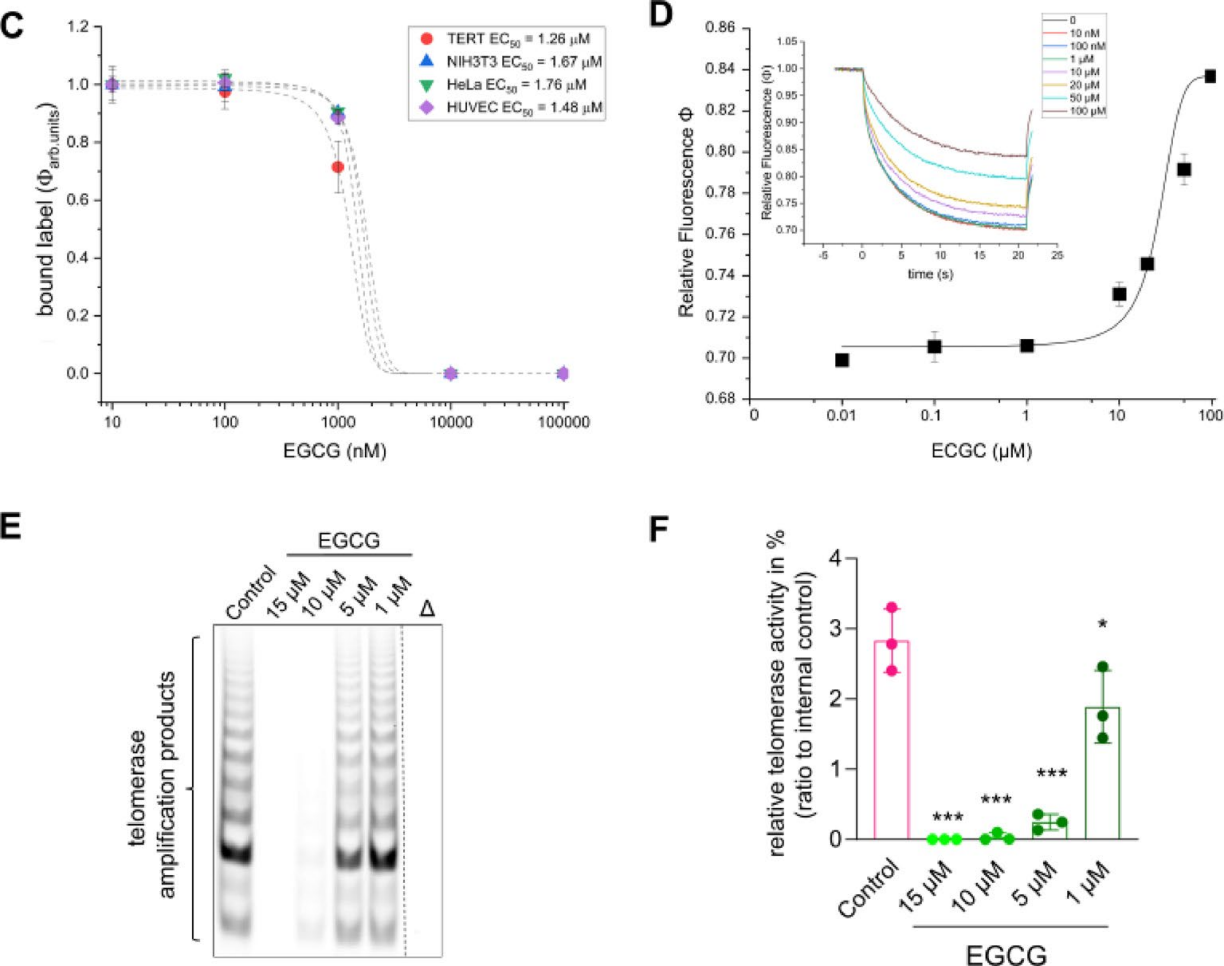
Evaluation of the binding affinity of epigallocatechin-3-gallate (EGCG) for TERT. (**A**) Structure of EGCG. (**B**) Microarray based binding assay monitoring TERT binding activity. The proteins or cell lysates were spotted in columns of ten dots. (left pads) EGCG series in presence of 100 nM Cy5-ATP; (right pads) EGCG series in presence of 10 nM primer cocktail. All binding experiments performed in binding buffer. The complete microarray is shown in Figure S5A, whereas in B is shown a section of six pads and in presence of 10 µM, 50 µM or 100 µM EGCG, respectively. (**C**) Histogram of fluorescence intensities (Φ) as a function EGCG concentration at proteins or cell lysates. The mean of the fluorescence intensities and standard deviation obtained from the microarray was used to estimate relative fluorescence as a function of different concentrations of EGCG from 0.01 to 100 µM. (**D**) Microscale thermophoresis (MST) experiment performed with TERT. Affinity of substances was performed with unlabelled TERT. Unlabelled TERT protein (30 µg) was incubated with primer cocktail and substance concentration as indicated overnight at 4° C. The ΔF data were normalized and fitted in the K_d_ model. (inset) Relative fluorescence as a function of time at different EGCG concentration. (**E**) Dose-dependent inhibition of cell lysate with high TERT activity in a TRAP assay by EGCG, observed across concentrations ranging from 1 to 15 µM. ∆ indicates heat inactivated cell lysate. (**F**) Quantification of respective TRAP samples. n = 3; *p < 0.05; ***p < 0.001; One-way ANOVA, Dunnett’s multiple comparisons test.

To validate the in-silico findings with a binding assay, we used several cell lines that express TERT; and human TERT obtained through recombinant expression in *E. coli* for the development of a highly miniaturized microarray-based nonradioactive TERT assay^24^. TERT was synthesized using pET28aTERT in *E. coli* and purified via Ni-IMAC (Figure S3). The TRAP assay confirmed the presence of TERT in NIH3T3 and HeLa cells, but not in HUVEC cells due to high passages (Fig. 2, Figure S4). For the microarray-based TERT binding assay, Hsp90α, purified TERT, and cell lysates were spotted onto nitrocellulose microarray slides in columns of ten dots as described earlier for other target-oriented tests on microarrays (Fig. 3). The results were evaluated using the same experimental system as reported in previous studies^42–47^. Hsp90α served as a control for nonspecific primer binding. The microarrays were incubated with a primer cocktail containing TERC, 5'-Cy5-TTAGGGTTAGGGTTAGGG-3' (Cy5-primer), Cy5-ATP, and dNTPs to enable pairing for enhanced binding (Fig. 3A). Only Cy5-ATP incubation showed ATP binding by human Hsp90α (Fig. 3B), whereas the addition of the primer cocktail led to fluorescence emission at all cell lysates and purified TERT (Fig. 3C). The advantage of this highly miniaturized TERT assay is the low material consumption, as between 800 and 1600 pL are used for a single spot at a Hsp90 concentration of 3 mg/mL. This would allow thousands of tests to be conducted from a single 50 µL cell lysate, for example. Another advantage is that the microarrays with Hsp90 are storable with printed proteins for at least one month. The microarray-based assay was utilized to test EGCG (Fig. 4A,B), a known TERT inhibitor^25^, by performing a dose-dependent titration using cell lysates or purified recombinant TERT at concentrations ranging from 0.01 to 100 µM (Figure S5A with the complete microarray). The main fluorescence signal originated from Cy5-primer, stabilized by the presence of TERC, Cy5-ATP, and dNTPs (Fig. 4B, Figure S5A with the complete microarray). For better visualization a section of three pads is shown in Fig. 4B. The binding of the fluorescence label was reduced as a function of EGCG concentration in a dose-responsive manner for purified TERT and cell lysates with an EC_50_ between 1.26 and 1.76 µM (Fig. 4C). The thermophoresis assay also estimated TERT’s affinity for EGCG, identifying a dose-responsive affinity with a *K*_*d*_ of ~ 26 µM (Fig. 4D). The observed difference in microarray-based TERT and thermophoresis assays may result from different accessibilities of the primer cocktail, in the microarray-based assay the proteins become fixed to the nitrocellulose surface. The TRAP assay further validated the inhibitory activity of EGCG, demonstrating dose-dependent inhibition within a range of 1 µM to 15 µM (Fig. 4E,F, Figure S6). This result corroborates the microarray assay’s screening capability for novel TERT-binding compounds.

**Fig. 5:**
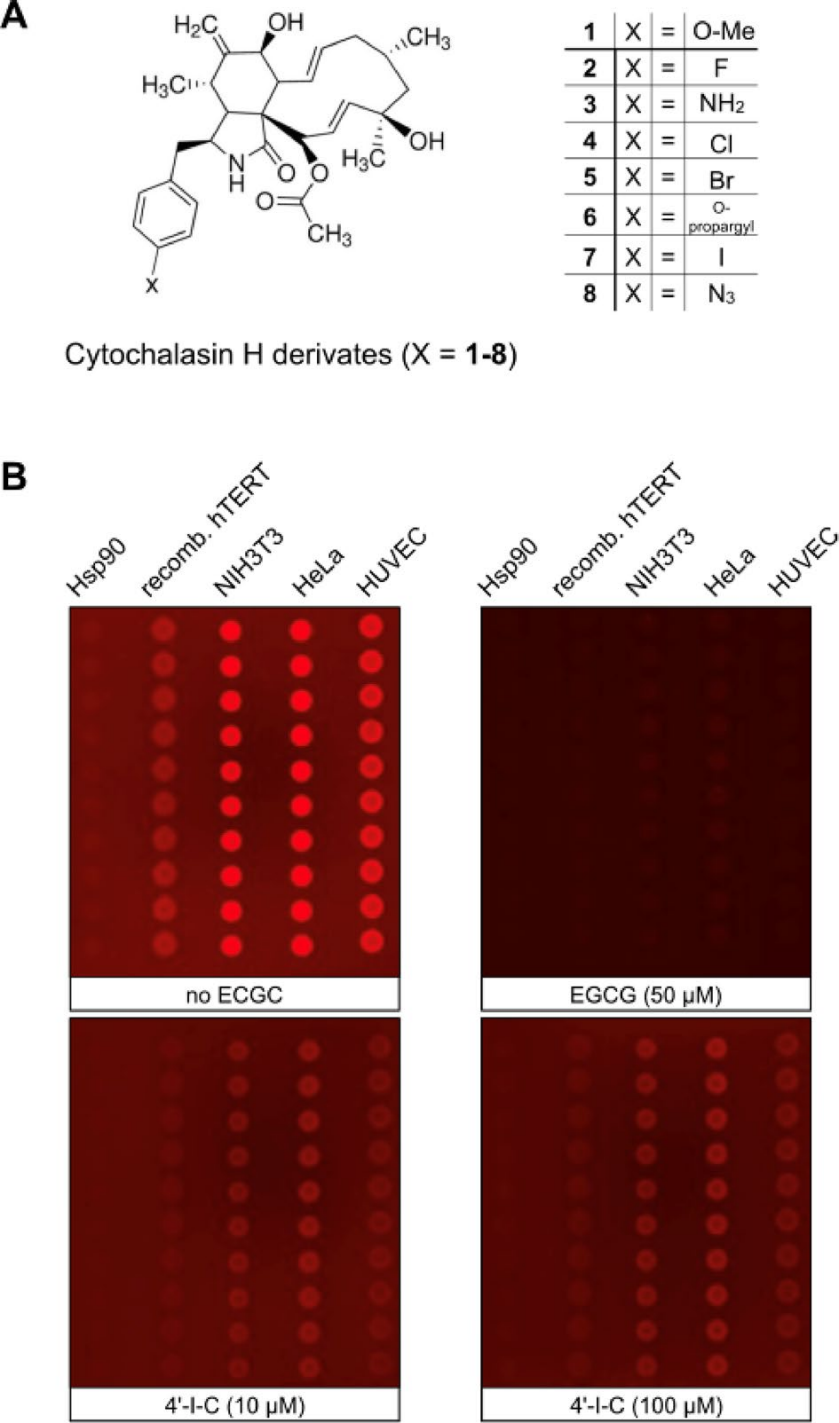

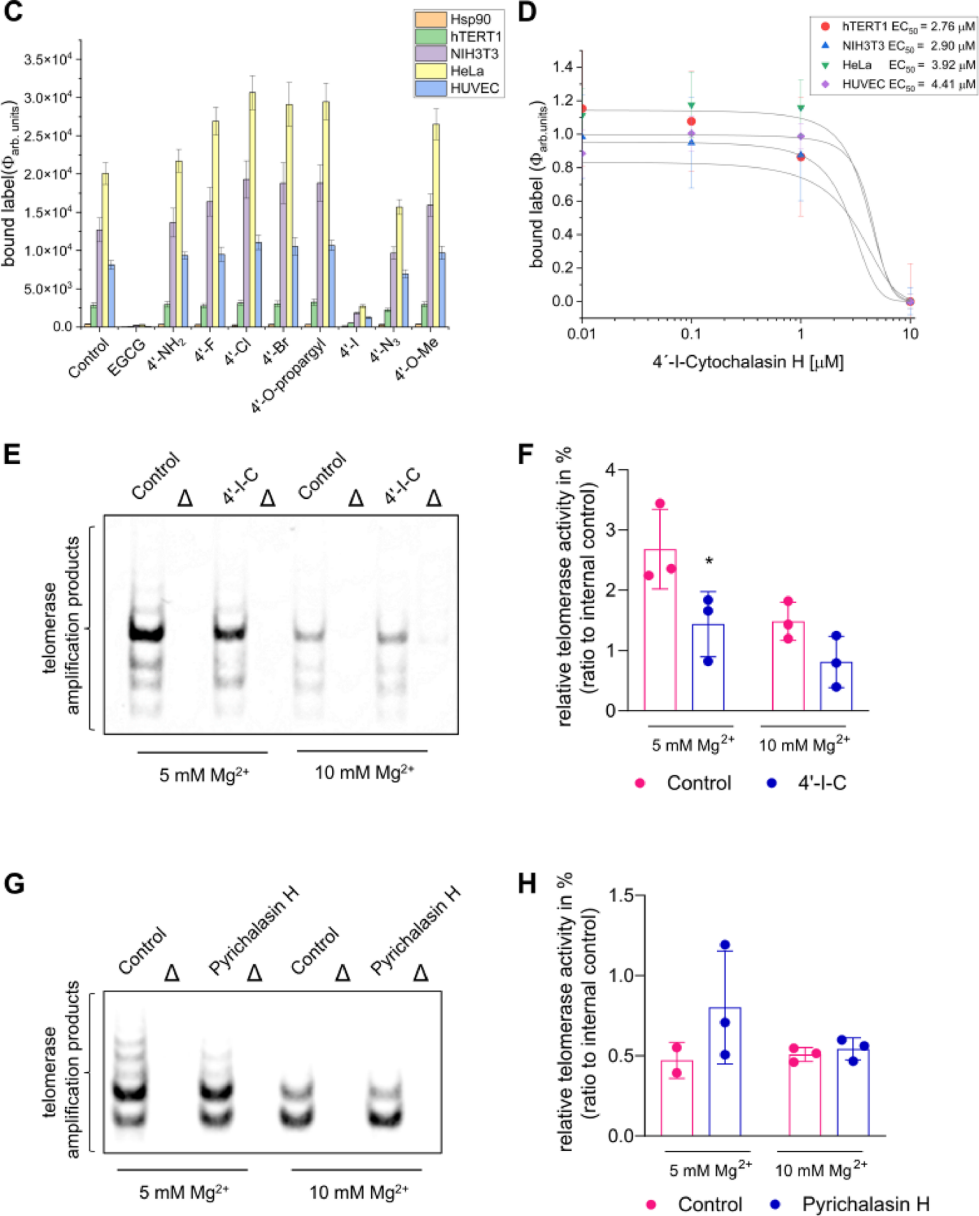
Microarray-based TERT binding activity in presence of cytochalasan derivatives. (**A**) Structures of cytochalasin-derivatives. Cytochalasin H and substitutions at position -R for 1 = 4´O-Me (Cytochalasin H), 2 = 4´-F, 3 = 4´-NH_2_, 4 = 4´-Cl, 5 = 4´-Br_,_ 6 = 4´-O-propargyl, 7 = 4´-I, 8 = 4´-N_3_. (**B**) The complete microarray is shown in Figure S5B, whereas in B is shown a section of four pads. The proteins are spotted in the same order as shown in Fig. 4. The first two pads of the microarray used for control without (left) and with 50 µM EGCG as a control for full displacement activity (right), followed by pads 3 and 4 with 10 µM incubation of 100 µM compound 7 in presence of 10 nM primer cocktail. (**C**) Bar histogram as the mean of the estimated of bound fluorescent intensities (Φ) as indicated on the chip measured with 100 µM compounds 1–8. (**D**) Displacement of fluorescence label was performed in presence of different concentrations of 4´-IC and fluorescence intensities (Φ) of bound Cy5-label of ten spots were used for estimation of the mean and dose response fittings. (**E**) High TERT activity in cell lysate could be attenuated in a TRAP assay by using 100 µM 4’-I-C and a higher Mg^2+^ concentration. (**F**) Quantification of respective TRAP samples. (**G**) Cytochalasin H could not inhibit the TERT activity in a TRAP assay by using the same condition as 4’-I-C. (**H**) Quantification of respective TRAP samples. ∆ indicates respective heat inactivated cell lysate. n = 3; *p < 0.05, Two-way ANOVA, Sidak’s multiple comparisons test.

Cytochalasins are among the supposedly well-studied natural products, but the potential targets they achieve have been only partially investigated. Therefore, cytochalasin derivatives were also tested on non-actin targets and the effect of cytochalasin H and its derivatives on primer binding was investigated. We therefore screened a small library of cytochalasin derivatives (Fig. 5A; R = 1–8) for TERT binding activity using the microarray assay. Most cytochalasin derivatives did not interfere with binding, except for 4´-iodo-cytochalasin H (4´-I-C). Evaluation of TERT inhibition monitored as bound fluorescence intensities revealed significant binding displacement by 4´-I-C at 10 µM (a section shown in Fig. 5B, Figure S5B-C with the complete microarray). The dose–response analysis showed an EC_50_ of 2.76–4.41 µM for 4´-I-C on TERT protein and cell lysates (Fig. 5C,D). In TRAP assays, 4´-I-C inhibited TERT activity at 5 mM Mg^2+^, but not at 10 mM Mg^2+^, suggesting that while a moderate increase in Mg^2+^ concentration enhances inhibition efficiency, excessively high concentrations may counteract this effect, potentially affecting telomerase activity itself (Fig. 5E,F, Figure S7A). Meanwhile, cytochalasin H, which lacks the 4’-iodine atom, was inactive under the same conditions (Fig. 5G,H, Figure S7B). We further examined the influence of divalent cations on 4´-I-C binding. Magnesium ions (Mg^2+^) significantly enhanced primer cocktail binding to TERT and cell lysates, followed by CoCl_2_, ZnCl_2_, and CaCl_2_. No binding was observed with MnCl_2_, SeNO_3_, and FeSO_4_, aligning with the requirement by insect telomerase for Mg^2+^ ions for DNA stabilization (Figure S8)^44^. Finally, simulated docking of 4’-I-C to TERT complex was achieved with DiffDock-L. A binding position near the DNA primer was identified. Binding of 4’-I-C at this position could potentially hinder the pairing to the TERC-RNA at TERT (Figure S9). The binding of 4’-I-C near the DNA binding site could explain the displacement of the Cy5 primer in the microarray and thermophoresis experiments.

## Discussion

This study highlights the versatility and potential of a microarray-based assay for the identification and characterization of new TERT inhibitors. The TERT binding assay was validated using the known inhibitor EGCG, and further screening of a small compound library of cytochalasins led to the discovery of 4’-I-C as a new TERT inhibitor. Both EGCG and 4’-I-C were shown to disrupt Cy5 primer cocktail binding in cell lysates as well as on purified TERT and the results from the binding assay were confirmed by the TRAP activity assay in cell lysate^25,26^. The newly developed assay format also holds promise for identifying potential TERT activators, which could enhance binding and TERT activity, potentially aiding targeted cellular regeneration^8,9,48,49^. Traditionally, cytochalasins exert their effects by binding to actin and preventing its polymerisation, but our findings shows that 4’-I-C, at least, has an additional non-actin target^27–29^.

High telomerase activity was confirmed in HeLa cell lysates using the TRAP assay, whereas HUVEC cells exhibited negligible TERT activity (Fig. 2). Interestingly, recombinant TERT expressed in *E. coli* showed binding activity in the microarray assay but lacked TRAP activity, indicating that additional cellular components or proper protein folding might be essential for full enzymatic function^50–55^. While this is a limitation, recombinant TERT remains valuable for studying compound interactions based on structural comparability. Among the tested samples, HeLa cell lysates demonstrated the highest TERT activity, suggesting the presence of necessary cofactors or proper protein conformation within these cells. The implications of these findings are multifaceted. The observed TERT inhibition by 4’-I-C expands the scope of telomerase inhibition strategies beyond traditional small molecules and nucleoside analogs against cancer^56,57^. Furthermore, the binding activities observed in HeLa cell lysates compared to recombinant TERT from *E. coli* emphasize the complexity of telomerase regulation, which likely involves additional protein cofactors or requirements for complex formation. Moreover, the presence of Mg^2^⁺ significantly enhanced the binding of the primer cocktail, whereas other divalent cations such as Mn^2^⁺, Se^4^⁺ and Fe^2^⁺ were unable to mediate or enhance the binding of the primer cocktail (Figure S8). An interesting aspect of our results is the differential response of the different cytochalasin derivatives, with only 4’-I-C exhibiting substantial TERT inhibition. The introduction of a subtle chemical modification such as the iodine atom led to a change in the binding affinity to TERT, and as shown by the microarray-based TERT assay, and particularly the TRAP activity assay, the presence of magnesium is responsible for the binding of the primer cocktail but also affects the binding of 4’-I-C. Here it can be proposed that an interaction between the iodine atom and magnesium at TERT is important for the displacement of the primer cocktail, since the other cytochalasins or other halogenated cytochalasins had no influence on the binding of the primer cocktail and cytochalasin H showed no influence on the TERT activity in the TRAP assay.

In conclusion, our study presents a robust and adaptable assay for screening TERT modulators, reaffirmed by the identification and characterization of novel inhibitors like 4’-I-C. The assay’s capability to detect subtle interaction dynamics and its applicability to high-throughput screening render it invaluable for ongoing and future research in telomerase biology and therapeutic development. The advantage of our microarray-based TERT assay is the high degree of miniaturization, combined with low material consumption (50 µL, 3 mg/mL for thousands of individual assays), and the combinatorial use allows different components to be tested comparatively. The novel insights into TERT regulation by divalent cations and the unique inhibition mechanism of 4’-I-C underscore the intricate regulatory landscape of telomerase, paving the way for innovative therapeutic interventions targeting telomerase-related pathologies. However, it is important to keep in mind that this microarray-based TERT assay primarily measures binding, which may not correlate with enzymatic inhibition. Unlike assays like TRAP, it does not measure activity or replicate the intracellular environment. Despite this limitation, the assay remains a valuable tool for identifying TERT-binding compounds prior to functional validation.

## Methods

### pET28a_TERT fusion construct

ATGGGCAGCAGCCATCATCATCATCATCACAGCAGCGGCCTGGTGCCGCGCGGCAGCCATATGCCACGTGCACCACGTTGTCGCGCAGTTCGTAGTCTGTTACGCAGCCATTATCGCGAAGTTCTGCCGTTAGCAACCTTTGTTCGTCGTCTGGGTCCACAAGGTTGGCGTTTAGTTCAACGTGGTGATCCAGCAGCATTTCGCGCTTTAGTTGCACAGTGCTTAGTTTGCGTTCCGTGGGATGCTCGTCCACCACCAGCAGCACCAAGTTTTCGTCAGGTAAGCTGCCTGAAAGAGCTGGTTGCACGCGTTTTACAACGTCTGTGCGAACGTGGCGCAAAAAACGTTCTGGCATTTGGTTTTGCGTTATTAGACGGCGCACGCGGCGGTCCACCAGAAGCATTTACCACCAGCGTTCGTAGCTATCTGCCAAATACCGTTACCGACGCATTACGCGGTTCAGGCGCTTGGGGTTTATTATTACGCCGCGTTGGCGACGACGTTTTAGTTCATCTGCTGGCACGTTGCGCGTTATTTGTTCTGGTTGCACCGAGTTGCGCATATCAAGTTTGCGGTCCACCGTTATATCAATTAGGCGCAGCAACCCAAGCACGTCCACCACCACACGCATCTGGTCCACGTCGTCGTTTAGGTTGCGAACGTGCTTGGAATCATAGCGTTCGCGAAGCAGGCGTTCCATTAGGTTTACCAGCACCAGGTGCACGTCGTCGCGGCGGTTCTGCAAGTCGTAGCTTACCGTTACCGAAACGTCCACGTCGTGGTGCAGCACCAGAACCAGAACGTACCCCAGTTGGTCAAGGTTCTTGGGCACATCCAGGTCGTACGCGCGGTCCATCTGATCGCGGTTTTTGTGTTGTTAGTCCGGCTCGTCCAGCAGAAGAAGCAACCAGCTTAGAAGGCGCATTAAGCGGTACCCGTCATAGTCATCCGTCTGTTGGTCGTCAACATCACGCAGGTCCACCAAGTACCAGTCGTCCACCACGTCCGTGGGATACCCCGTGTCCACCGGTTTACGCGGAAACCAAACATTTCCTGTACAGCAGCGGCGATAAAGAACAGCTGCGTCCGAGTTTTCTGCTGAGTAGCTTACGTCCAAGTTTAACCGGCGCACGTCGTTTAGTCGAAACCATTTTTCTGGGTTCTCGTCCGTGGATGCCAGGTACCCCACGTCGTTTACCACGTTTACCACAGCGTTACTGGCAGATGCGTCCGTTATTTCTGGAACTGCTGGGCAATCACGCACAATGTCCATACGGCGTTCTGCTGAAAACCCATTGTCCATTACGCGCAGCAGTTACCCCAGCAGCAGGCGTTTGCGCACGCGAAAAACCACAAGGTTCTGTTGCAGCACCGGAAGAAGAAGATACCGATCCACGTCGTCTGGTTCAGTTACTGCGTCAACATTCTAGTCCGTGGCAGGTTTACGGTTTTGTTCGCGCTTGTCTGCGTCGTTTAGTTCCACCAGGTTTATGGGGTAGCCGTCATAACGAACGTCGTTTTCTGCGCAACACCAAAAAATTCATCAGCCTGGGCAAACACGCAAAACTGAGCCTGCAGGAATTAACCTGGAAAATGTCCGTCCGCGATTGCGCTTGGTTACGTCGTTCTCCAGGCGTTGGTTGCGTTCCAGCAGCGGAACATCGTTTACGCGAAGAAATCCTGGCGAAATTTCTGCACTGGCTGATGAGCGTGTACGTTGTTGAACTGCTGCGTAGCTTCTTCTACGTCACCGAAACCACCTTCCAGAAAAACCGCCTGTTCTTCTACCGCAAAAGCGTTTGGAGCAAACTGCAGAGCATTGGTATCCGTCAGCATCTGAAACGCGTTCAACTGCGCGAATTAAGCGAAGCGGAAGTTCGTCAACATCGCGAAGCACGTCCAGCATTATTAACCAGCCGTCTGCGTTTCATTCCGAAACCAGACGGCTTACGTCCAATCGTCAACATGGACTACGTCGTTGGTGCACGTACCTTTCGTCGCGAAAAACGCGCAGAACGTTTAACCAGTCGCGTTAAAGCGCTGTTCAGCGTTCTGAATTACGAACGCGCACGTCGTCCAGGTTTATTAGGCGCAAGCGTTCTGGGTTTAGACGATATTCATCGCGCCTGGCGTACCTTTGTTTTACGCGTTCGCGCACAAGATCCACCGCCGGAACTGTATTTCGTCAAAGTCGATGTTACCGGCGCATACGATACCATTCCACAAGATCGTCTGACCGAAGTTATTGCGAGCATCATCAAACCGCAGAACACCTATTGCGTTCGCCGTTACGCAGTTGTTCAGAAAGCAGCGCACGGTCACGTTCGTAAAGCGTTTAAAAGCCATGTTAGCACCCTGACCGATTTACAGCCGTATATGCGCCAGTTTGTTGCGCATCTGCAAGAAACCAGCCCATTACGCGACGCAGTTGTTATCGAACAGAGCAGCAGCCTGAACGAAGCATCTAGCGGCCTGTTTGACGTTTTTCTGCGCTTCATGTGCCATCACGCAGTTCGTATTCGCGGTAAATCCTACGTTCAGTGTCAGGGTATTCCGCAAGGTAGCATTCTGTCTACCCTGTTATGCAGCCTGTGCTACGGCGATATGGAGAACAAACTGTTTGCGGGTATTCGTCGCGACGGTTTACTGCTGCGTCTGGTCGACGATTTTCTGCTGGTTACCCCGCATTTAACCCACGCAAAAACCTTTCTGCGTACCTTAGTTCGCGGCGTTCCAGAATACGGCTGCGTTGTTAACCTGCGTAAAACCGTCGTCAACTTCCCGGTTGAAGATGAAGCGTTAGGCGGTACCGCTTTTGTTCAGATGCCAGCACACGGTTTATTTCCGTGGTGCGGTTTACTGTTAGATACCCGTACCCTGGAAGTCCAGAGCGATTATAGCAGCTACGCACGTACCAGTATTCGCGCAAGTCTGACCTTTAACCGCGGTTTTAAAGCCGGCCGTAATATGCGTCGTAAACTGTTTGGCGTCTTACGTCTGAAATGCCACAGCCTGTTCCTGGATCTGCAAGTTAACAGCCTGCAGACCGTTTGTACCAACATCTACAAAATCCTGCTGCTGCAGGCCTACCGTTTTCACGCTTGCGTTTTACAGCTGCCGTTTCATCAGCAGGTCTGGAAAAACCCGACCTTCTTTCTGCGCGTTATTAGCGATACCGCCAGCCTGTGTTACAGCATCCTGAAAGCGAAAAACGCAGGTATGAGTTTAGGCGCAAAAGGCGCAGCAGGTCCATTACCATCTGAAGCTGTTCAGTGGCTGTGTCATCAGGCGTTTCTGTTAAAACTGACCCGCCATCGCGTTACCTACGTTCCATTACTGGGTAGTTTACGTACCGCACAGACCCAACTGAGTCGTAAATTACCGGGTACCACCCTGACCGCATTAGAAGCAGCAGCAAATCCGGCACTGCCGTCTGATTTCAAAACCATTCTGGACTAA.

### TERT fusion protein

MGSSHHHHHHSSGLVPRGSHMPRAPRCRAVRSLLRSHYREVLPLATFVRRLGPQGWRLVQRGDPAAFRALVAQCLVCVPWDARPPPAAPSFRQVSCLKELVARVLQRLCERGAKNVLAFGFALLDGARGGPPEAFTTSVRSYLPNTVTDALRGSGAWGLLLRRVGDDVLVHLLARCALFVLVAPSCAYQVCGPPLYQLGAATQARPPPHASGPRRRLGCERAWNHSVREAGVPLGLPAPGARRRGGSASRSLPLPKRPRRGAAPEPERTPVGQGSWAHPGRTRGPSDRGFCVVSPARPAEEATSLEGALSGTRHSHPSVGRQHHAGPPSTSRPPRPWDTPCPPVYAETKHFLYSSGDKEQLRPSFLLSSLRPSLTGARRLVETIFLGSRPWMPGTPRRLPRLPQRYWQMRPLFLELLGNHAQCPYGVLLKTHCPLRAAVTPAAGVCAREKPQGSVAAPEEEDTDPRRLVQLLRQHSSPWQVYGFVRACLRRLVPPGLWGSRHNERRFLRNTKKFISLGKHAKLSLQELTWKMSVRDCAWLRRSPGVGCVPAAEHRLREEILAKFLHWLMSVYVVELLRSFFYVTETTFQKNRLFFYRKSVWSKLQSIGIRQHLKRVQLRELSEAEVRQHREARPALLTSRLRFIPKPDGLRPIVNMDYVVGARTFRREKRAERLTSRVKALFSVLNYERARRPGLLGASVLGLDDIHRAWRTFVLRVRAQDPPPELYFVKVDVTGAYDTIPQDRLTEVIASIIKPQNTYCVRRYAVVQKAAHGHVRKAFKSHVSTLTDLQPYMRQFVAHLQETSPLRDAVVIEQSSSLNEASSGLFDVFLRFMCHHAVRIRGKSYVQCQGIPQGSILSTLLCSLCYGDMENKLFAGIRRDGLLLRLVDDFLLVTPHLTHAKTFLRTLVRGVPEYGCVVNLRKTVVNFPVEDEALGGTAFVQMPAHGLFPWCGLLLDTRTLEVQSDYSSYARTSIRASLTFNRGFKAGRNMRRKLFGVLRLKCHSLFLDLQVNSLQTVCTNIYKILLLQAYRFHACVLQLPFHQQVWKNPTFFLRVISDTASLCYSILKAKNAGMSLGAKGAAGPLPSEAVQWLCHQAFLLKLTRHRVTYVPLLGSLRTAQTQLSRKLPGTTLTALEAAANPALPSDFKTILD*

### TERC RNA sequence

UAAUACGACUCACUAUAGGGAGAGGGUUGCGGAGGGUGGGCCUGGGAGGGGUGGUGGCCAUUUUUUGUCUAACCCUAACUGAGAAGGGCGUAGGCGCCGUGCUUUUGCUCCCCGCGCGCUGUUUUUCUCGCUGACUUUCAGCGGGCGGAAAAGCCUCGGCCUGCCGCCUUCCACCGUUCAUUCUAGAGCAAACAAAAAAUGUCAGCUGCUGGCCC

### Cy5-primer sequence. 5´-Cy5- TTAGGGTTAGGGTTAGGG-3´

#### Cell cultivation and immune blot analysis

Murine fibroblasts (NIH3T3, ATCC® CRL-1658TM), HeLa cells and HUVEC cells were cultured in DMEM (Dulbecco’s Modifed Eagle Medium, Bio&Sell BS.FG 0445) containing 10% FCS (Bio&Sell BS.L 2045) and 1% Penicillin–Streptomycin-Mix (Bio&Sell BS-AB17.07001) at 37 °C in a humidified environment with 5% CO_2_. The culture was harvested at the designed time intervals by trypsin (0.02% EDTA included) then was washed with Hank’s Buffered Salt Solution (Bio&SELL BS.L 2045). The cell lysates were subsequently generated by adding glass beads and sonicated in buffer (20 mM Tris–HCl pH 8.0, 500 mM KCl, 2 mM β-Mercaptoethanol, 2 mM Imidazole, 10% Glycerin, 1% protease inhibitors (Carl Roth 3751.1), 10 mM Dithiothreitol for 10 s and 6 times. The lysates were centrifugated at a speed of 17,000 × g for 10 min at 4 °C and the supernatant was analyzed by separation on SDS-PAGE and immune detection was performed with anti-telomerase (biorbyt, orb515178) and anti-mouse alkaline phosphatase secondary antibody (Sigma-aldrich A1047).

#### Preparation of recombinant human Hsp90a and TERT

Hsp90α was recombinantly synthesized and purified as described recently^38^. The DNA of TERT was obtained as a codon usage adapted clone from Synbio Technologies LLC, USA provided in pET28a plasmid (DNA Sequence and corresponding protein sequence with the tag in grey). Afterwards, the pET28aTERT construct was transferred into *E. coli* BL21 Arctic express cells for the preparation of recombinant protein. *E. coli* cultures were induced after growing at 37 °C for 8 h by 1 mM IPTG at 8 °C for 72 h. The cells containing the pET28aTERT construct were sedimented at 8,500 × g for 15 min and disrupted in 20 mM Tris pH 8.0, 137 mM NaCl, 10% glycerin, and further lysis of the cells was generated by two cycles in a French cell press between 14.000–16,000 psi. The lysate was centrifugated, the supernatant was removed, and the sediment was solubilized in 20 mM Tris pH 8.0, 137 mM NaCl, 8 M urea and 1% *N*-lauroylsarcosine for 1 h. The solution was centrifugated at 15.000xg for 30 min. The supernatant was diluted fourfold and TERT was purified on a Ni-IMAC column (Cube Biotech 74,306). Bound His-tagged TERT produced by affinity column was finally eluted and concentrated to 3 mg/ml into buffer containing 20 mM Tris pH 7.5, 50 mM NaCl, 2 mM β-mercaptoethanol, 10% glycerin using Amicon (30 K) centrifugal concentrators (Merck Millipore C7715). The presence and purity of TERT was detected by SDS-PAGE and immune blotting using anti-His antibody (biorbyt, orb238446) and secondary anti-rabbit alkaline phosphatase (Sigma-Aldrich A8025).

#### Detection of microarray-based Hsp90 or TERT binding activity

Purified full-length human Hsp90α, recombinant TERT or cell lysates were transferred into 20 mM Tris–HCl, pH 7.5, 50 mM KCl, 6 mM ß-mercaptoethanol, 10% (*v/v*) glycerol and spotted on the UniSart® 3D nitro slide (Sartorius Stedim Biotech S.A. 2,000,125) using a contactless GeSim Nano-PlotterTM (GeSim) with a nanotip pipette as described earlier using a protein concentration of 3 mg/mL^38^. Columns of ten spots of proteins or lysates were spotted in binding buffer containing Hepes 20 mM, KCl 50 mM, 5 mM MgCl_2_.6H_2_O, 0.01% Tween 20, 0.1 mg/mL BSA, 1 mM DTT using a contactless Plotter (Gesim Nano Plotter) on a nitrocellulose coated glass slide. The microarray slides were transferred into an incubation chamber and incubated in blocking buffer (Candor blocking solution). Microarray slides were fixed in the incubation chamber. To each pad, 100 µL of the blocking buffer was added. Incubation was carried out for 45 min on a shaker at room temperature. For ATP-binding assay, the slides were incubated directly with 100 nM dye labelled ATP in binding buffer (20 mM HEPES–KOH, pH 7.3, 50 mM KCl, 5 mM MgCl_2_, 20 mM Na_2_MoO_4_, 0.01% (*v/v*) Tween 20, 2% (*v/v*) DMSO, 0.1 mg/ml BSA) for 16 h at 4 °C. The buffer was discarded, and slide was removed from the incubation chamber. The pads were completely dried with compressed air. The slide was scanned at excitation wavelength of 635 nm excitation wavelength, laser power 10%, PMT gain 380 of the laser using a GenePrix scanner and calculated with ImaGene 5 of BioDiscovery, Inc. Evaluation of displacement was done with the EC_50_ value. The dose–response curves were calculated with Origin 7G (OriginLab Corporation) and fitted with the non-linear function logistic, A1 = 0, A2 = 1. Quality validation of the microarray performed by calculating the mean (± s.d.) of 10 spots as described before.

For TERT activity 5´-Cy5-TTAGGGGTTAGGGTTAGGG-3’ primer obtained from (Eurofins genomics), TERC-RNA synthesized from TERC DNA (Synbio Technologies LLC, USA) by T7 RNA polymerase and purified as described earlier^34^. Proteins or cell lysates spotted on slides as described before were incubated with blocking solutions and afterwards 10 nM Cy5-labelled primer, 2 µg/mL TERC, 10 nM ATP-Cy5 and 100 nM dNTPs were used in incubation buffer at 4 °C for 16 h. Afterwards the solution was washed by binding buffer containing 20 mM Hepes, 50 mM KCL, 5 mM MgCl_2_, 0.01% Tween 20, 0.1 mg/mL BSA, 1 mM DTT, dried and scanned as described before.

#### Microscale thermophoresis (MST) experiments

Recombinant TERT was dialyzed against 100 mM sodium bicarbonate buffer pH 8.5 at 4 °C for 16 h. Affinity of substances was performed with unlabelled TERT. Unlabelled TERT protein (30 µg) was incubated with 100 ng TERC, 10 nM Cy5-TTAGGGGTTAGGGTTAGGG-3’, 100 nM dNTP, 10 nM Cy5-ATP and substance concentration as indicated overnight at 4 °C. The labelled TERT proteins were dissolved in DMSO, and seven 1:5 dilutions were prepared in DMSO. Each final MST sample contained 50 nM. TERT and inhibitors in concentrations ranging from 100 µM to 10 nM, yielding a final DMSO concentration of 2.5%. The MST measurement was performed using a NanoTemper Monolith NT.115 G008, 20% excitation power and 40% MST power.

#### Purification of cytochalasin derivatives

Cytochalasin derivatives were isolated from *M. grisea* NI980 strain as described earlier^27^. The extraction procedure started with *M. grisea* spores using a pyiA knock-out strain in order to feed the cultures with precursor like para chloro- /bromo-^27,28^. Fermentation, metabolite preparation and characterisation of the derivative iodo-phenylalanine was described according to^28^ and characterised after HPLC purification. Purity and structure were determined by NMR spectroscopy (Figure S10).

#### Strains and culture conditions

*M. grisea ∆*pyiA^[28]^ as cultivated on Oatmeal Agar (OMA) (4% *w/v* oat meal, 0.5% *w/v* sucrose, 2% agar) or Complete Medium (CM) agar at 25 °C. For cytochalasan production, strains were cultivated in Soy Sauce Sucrose (SSS) medium (5% *v/v* soy sauce, 5% *w/v* sucrose, in autoclaved tap water) or DPY liquid medium (2% *w/v* dextrin from potato starch, 1% *w/v* polypeptone, 0.5% *w/v* yeast extract, 0.5% *w/v* monopotassium phosphate, 0.05% *w/v* magnesium sulfate, 2.5% *w/v* agar) for 7 days at 25 °C, 110 rpm.

#### Fermentation and extraction protocols

For extraction, *M. grisea ∆pyiA* spores were collected from MG agar plates incubated for 7 days and inoculated into 500 mL Erlenmeyer flasks containing 100 mL DPY. The spores were allowed to grow in the liquid culture for 7–8 days on shakers at 110 rpm at 25 °C. The cells were blended in the fermentation broth, filtered to remove the mycelium, and transferred into a separating funnel. An equal volume of EtOAc was added into the separating funnel and the aqueous layer was extracted twice. The organic solvent from the two extractions was combined and dried with MgSO_4_, filtered and evaporated to dryness. The crude extract was dissolved into 1 mL HPLC grade MeOH.

#### Preparation of 4’-iodocytochalasin H

200 mg of *para*- iodo-phenylalanine (Sigma-Aldrich**)** was dissolved into double distilled water (final concentration: 10 mg/mL), stirred at 60 ˚C until dissolved completely and filter-sterilized. 200 µl of this solution was fed to the *M. grisea ∆pyiA* inoculated into 500 mL Erlenmeyer flasks containing 100 mL DPY. The feeding was repeated at 24 h intervals four times. On the 7^th^ day the cultures were extracted using the method described above.

#### Analytical LCMS

LCMS data were obtained using a Waters LCMS system comprising of a Waters 2767 autosampler, Waters 2545 pump system and a Phenomenex Kinetex column (2.6 µ, C_18_, 100 Å, 4.6 × 100 mm) equipped with a Phenomenex Security Guard precolumn (Luna C_5_ 300 Å) eluted at 1 mL/min. Detection was performed by Waters 2998 diode array detector between 200 and 600 nm; Waters 2424 ELSD and Waters SQD-2 mass detector operating simultaneously in ES^+^ and ES^-^ modes between 100 m/z and 650 m/z. Solvents were A, HPLC-grade H_2_O containing 0.05% formic acid; and B, HPLC-grade CH_3_CN containing 0.045% formic acid. Gradients were as follows: Method 1 (optimised for non-polar compounds): 0 min, 10% B; 10 min 90% B; 12 min, 90% B; 13 min, 10% B and 15 min, 10% B. Method 2 (optimised for polar compounds): 0 min, 10% B; 10 min 40% B; 12 min, 90% B; 13 min, 10% B and 15 min, 10% B.

#### Preparative LCMS

Compounds were purified using a Waters massdirected autopurification system consisting of a Waters 2545 pump and Waters 2767 autosampler. The chromatography column was a Phenomenex Kinetex Axia column (5μ, C_18_, 100 Å, 21.2 × 250 mm) fitted with a Luna C_5_ 300 Å Phenomenex Security Guard precolumn. The column was eluted at 20 mL/min at 22 °C. Solvents used were A, H_2_O + 0.05% formic acid; and B, CH_3_CN + 0.045% formic acid. All solvents were HPLC grade. The column outlet was split (100:1) and the minority flow was supplemented with HPLC-grade MeOH + 0.045% formic acid to 1000 μL/min and diverted for interrogation by diode array (Waters 2998) and evaporative light-scattering (Waters 2424) detectors. The flow was also analysed by mass spectrometry (Waters SQD-2 in ES^+^ and ES^−^ modes). Desired compounds were collected into glass test tubes. Combined fractions were evaporated *in vacuo*, then dissolved directly in HPLC-grade MeOH to make the final concentration 10 mg/mL, 300 μl solution was loaded into the columnfor purification.

#### HRMS

HRMS was obtained using a UPLC system (Waters Acquity Ultraperformance, running the same method and column as above) connected to a Q-TOF Premier mass spectrometer.

#### NMR

A Bruker Avance 500 instrument equipped with a cryo-cooled probe at 500 MHz (^1^H)/125 MHz (^13^C) and 600 MHz (^1^H)/150 MHz (^13^C) were used for all NMR analysis. Standard parameters were used for the collection of 2D spectra (^1^H, ^1^H-correlation spectroscopy [COSY], heteronuclear single-quantum coherence [HSQC] and HMBC) in the indicated solvents. 1H and 13C spectra are referenced relative to residual protonated solvents. All δ values are quoted in ppm and all* J* values in Hz.

#### Developing of structural TERT models and docking experiments

Machine learning-based structural models of human TERT was generated using Alphafold3 using the TERT DNA sequence including TERC RNA sequence, DNA primer sequence and ATP and 10 magnesium ions. The TERT and the structural homologue of the insect telomerase (pdb: 6USO) was used for Diff-Dock-L experiments with EGCG, cytochalasin H and 4´-iodo-cytochalasin H^37^.

#### TRAP

The previously described TRAP assay was adapted and modified^42^. Certain amount of live cells were collected in 1.5 mL nuclease-free Eppendorf tubes and spun down at 5000×*g* for 5 min at 4 °C. After discarding the supernatant, the cell pellet was snap frozen in liquid nitrogen and stored at − 70 °C until needed. NP-40 lysis buffer (0 ˚C) was used to resuspend the cell pellet to a concentration of 1,000 cells/µL. The lysis was incubated for 30 min at 0 °C. For the heat inactivated samples, lysate was incubated for 10 min at 85 °C. 1 µL lysate and was added to 24 µL of master mix with the respective concentration of compound. The TRAP master mix contains 50X primer Mix (100 ng/mL each of ACX and NT primers along with 0.001 attomol/mL TSNT primers), DY-682 labelled TS primer (Table S1) and the respective compound. The inhibitor was added to the lysate rather than treating cells to prevent degradation or loss during harvesting and processing, which ensured direct interaction with telomerase. In the control samples the vehicle was added. After the TRAP-PCR protocol ((25 °C for 30 min; 95 °C for 5 min; 24 cycles of 95 °C for 30 s, 52 °C for 30 s, 72 °C for 40 s) 10 µL of each sample was loaded into a 10% acrylamide gel (19:1 Acryl:Bis acrylamide in Tris–borate-EDTA buffer) and resolved for around 2 h at 250 V and 4 °C in 0.5X Tris–borate-EDTA buffer. Then the gel was transferred to a fixative (0.5 M NaCl, 50% EtOH, 40 mM NaAc at pH 4.2) until it was scanned using LICORBio™ Odyssey.

## Supplementary Information

Supplementary Information.

## Data Availability

Should any raw data files be needed in another format they are available from the corresponding author upon reasonable request.
