# Supplementary material for: Development of a microarray based telomerase binding assay reveals unusual binding of a cytochalasin derivative
Source: Sci Rep. 2025 Jun 4;15:19515. doi: 10.1038/s41598-025-00230-z (PMC12137546; doi:10.1038/s41598-025-00230-z)
Supplement: Supplementary file 1 — Supplementary Information. [file 41598_2025_230_MOESM1_ESM.pdf]

Development of a Microarray Based Telomerase Binding Assay Reveals Unusual Binding of a Cytochalasin Derivative

Jia Li Ye[1, +], Lu Fan[3,+], Christian Bär [1,2], Thomas Thum[1], Oliver Plettenburg [3,4], Russell J. Cox[3], and Carsten Zeilinger[3*]

Supporting information

Fig. S1: Structural model of TERT (TERT1.pdb) modeled by AlphaFold3 with TERC-RNA, DNA primer, Mg-ATP and magnesium. TERT model is shown as a cartoon (green), and TERC-RNA as cartoon (green and orange) and the single stranded DNA primer is shown as a surface cartoon (cyan) and magnesium as green balls.

Fig. S2: Visualization of the putative binding sites of EGCG at TERT. A) Diff-Dock-L analysis of putative binders of EGCG using rank as a function of SMINA affinity using for EGCG an isomeric SMILES(C1[C@H]([C@H](OC2=CC(=CC(=C21)O)O)C3=CC(=C(C(=C3)O)O)O)OC(=O)C4=CC(=C(C(=C4)O)O)O) and the crystal structure obtained with values greater zero are given in blue, while values smaller than zero in red. **B)** Binding position of EGCG in the structure of human TERT (blue) (21) with several positions for EGCG near the ATP-binding site, **C)** Two position of EGCG in the TERT model neighboring to the adenine ring of ATP (yellow EGCG; -3.6 kcal/mole) or phosphate backbone (white EGCG; -2.8 kcal/mole).

Fig. S3: Immune detection of recombinant purified human TERT. A) The Ni-IMAC purified human TERT was separated by SDS-PAGE and were immune decorated after blotting with anti-TERT antibody.

Fig. S4: TRAP for detection of telomerase activity. Respective full-length TRAP assay blot with internal control. Different concentration of recombinant hTERT isolated from *E. coli* and purified with Ni-IMAC, HeLa, NIH3T3 and HUVEC at passage 3 (P3) and 9 (P9) cell lysates is shown. Δ indicates corresponding heat inactivated cell lysate.

Fig. S5: Microarray-based TERT binding activity in the presence of EGCG concentration series, cytochalasan derivatives (2-8) or concentration series of 4'-I-C (7). A) Microarray-based TERT binding activity in the presence of EGCG concentration series. The left panels of the microarray were tested in the presence of 100 nM Cy5-ATP and the right panels with 10 nM primer cocktail, while the first 2 panels serve as control, the remaining panels were tested with EGCG concentration series between 10 nM and 100 µM, respectively at

indicated concentrations. **B)** Microarray-based TERT binding activity in the presence of cytochalasan derivatives (Fig. 5A, **1-8**). Binding of the primer cocktail (10 nM) was determined with and without compounds. The first upper row of pads serves as a control. The first upper left pad of the microarray serves as a control with 10 nM primer cocktail and the upper right pad was incubated with 10 nM primer cocktail and 50 µM EGCG. Cytochalasan derivatives (**1-8**) are tested in the other pad rows 2-8. In pad row 2, concentrations of 100 µM derivates **3** and **2** are tested, respectively, while the other left-hand pad rows are tested with 10 µM and on the right-hand pad row with 100 µM for the other derivatives **3-8**. **C)** Concentration dependent titration of 4´-IC (**7**) in presence of Cy5-ATP (left pad row) or Cy5-primer cocktail (right pad row).

Fig. S6: TRAP assay for EGCG validation. Respective full-length TRAP assay blot. Dose-dependent inhibition of cell lysate with high TERT activity in a TRAP assay by EGCG, observed across concentrations ranging from 1 to 15 µM. Δ indicates heat inactivated cell lysate.

Fig. S7: TRAP assay to validate cytochalasan derivates. A) Respective full-length TRAP assay blot with internal control. High TERT activity in cell lysate could be attenuated in a TRAP assay by using 100 µM 4'-I-C and a higher Mg^{2+} concentration. **B)** Respective full-length TRAP assay blot with internal control. Cytochalasin H could not inhibit the TERT activity in a TRAP assay by using the same condition as 4'-I-C.

Fig. S8: Influence of additives as determined by the microarray based TERT assay. TERT or cell lysates were spotted in rows of ten spots to determine the mean value of fluorescence intensities. The binding activity of the primer cocktail was determined in the presence of 10 mM additives ($MnNO_3$, $SeNO_3$, $CoCl_2$, $MgCl_2$, $ZnCl_2$, $FeSO_4$, $CaCl_2$) and the mean values of bound fluorescence intensities (Φ) of purified Hsp90 and TERT and cell lysates from NIH3T3 cells, HeLa cells and HUVEC cells were plotted as a bar histogram.

Fig. S9: Visualization of the putative binding sites of 4´-IC at TERT using Diff-Dock software. Structural model of TERT (wheat) in presence of single stranded DNA-primer (stick yellow, orange) with 4´-IC (magenta), ATP (blue) and magnesium (green balls). For 4´-iodo cytochalasin H the SMILES code ([H]C1C(=C)[C@@H](O)[C@@H]2\C=C\CC([H])C[C@@]([H])(O)\C= C\C(OC(C)=O)[C@]22C(=O)N[C@@H](CC3=CC=C(I)C=C3)[C@]12[H]) was used.

Fig. S10: NMR data of 4´-iodocytochalasin H recorded at 400 MHz in CDCl3. Values are in agreement with published data.

Table S1: List of primers used in TRAP Assay

Primer	Sequence (5' → 3')

TSNT	AATCCGTCGAGCAGAGTTAAAAGGCCGAGAAGCGAT
NT	ATCGCTTCTCGGCCTTTT
ACX	GCGCGGCTAACCCTAACCCTAACC
DY-682 labelled TS	AATCCGTCGAGCAGAGTT

Supplement Figure 1

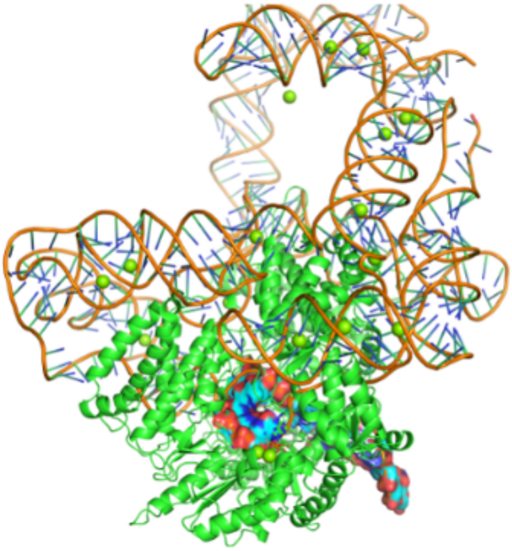

Supplement Figure 2

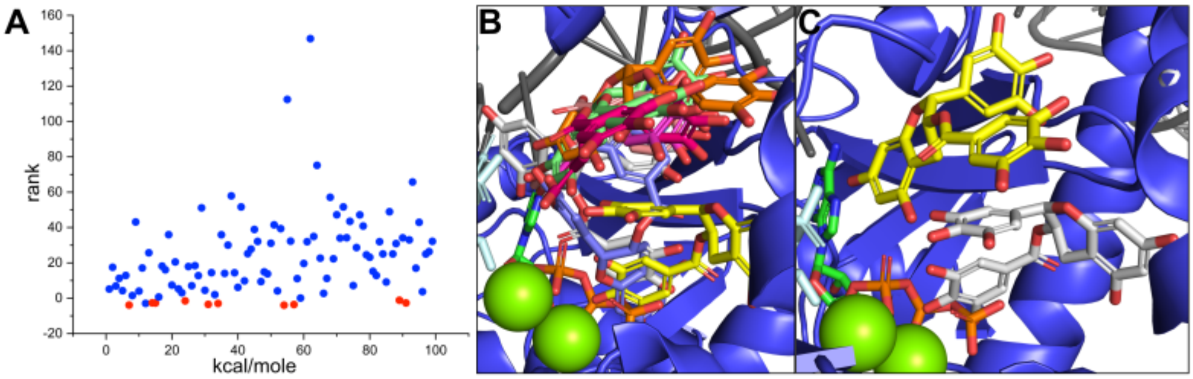

Supplement Figure 3

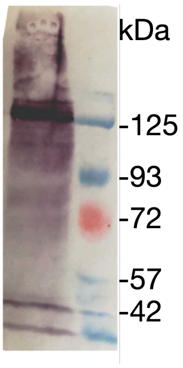

Supplement Figure 4

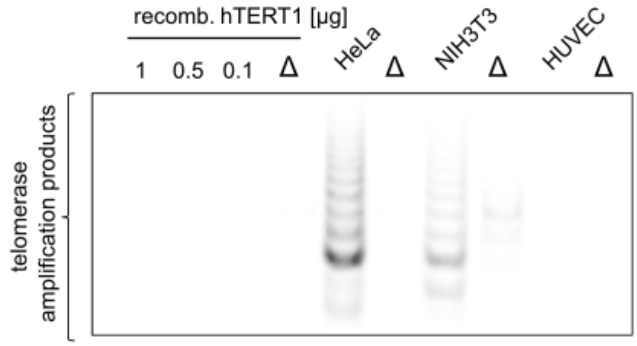

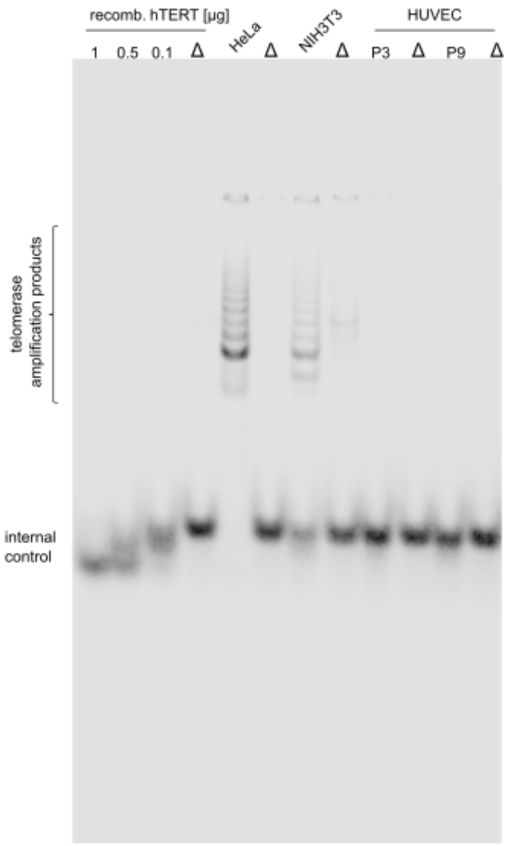

Supplement Figure 5

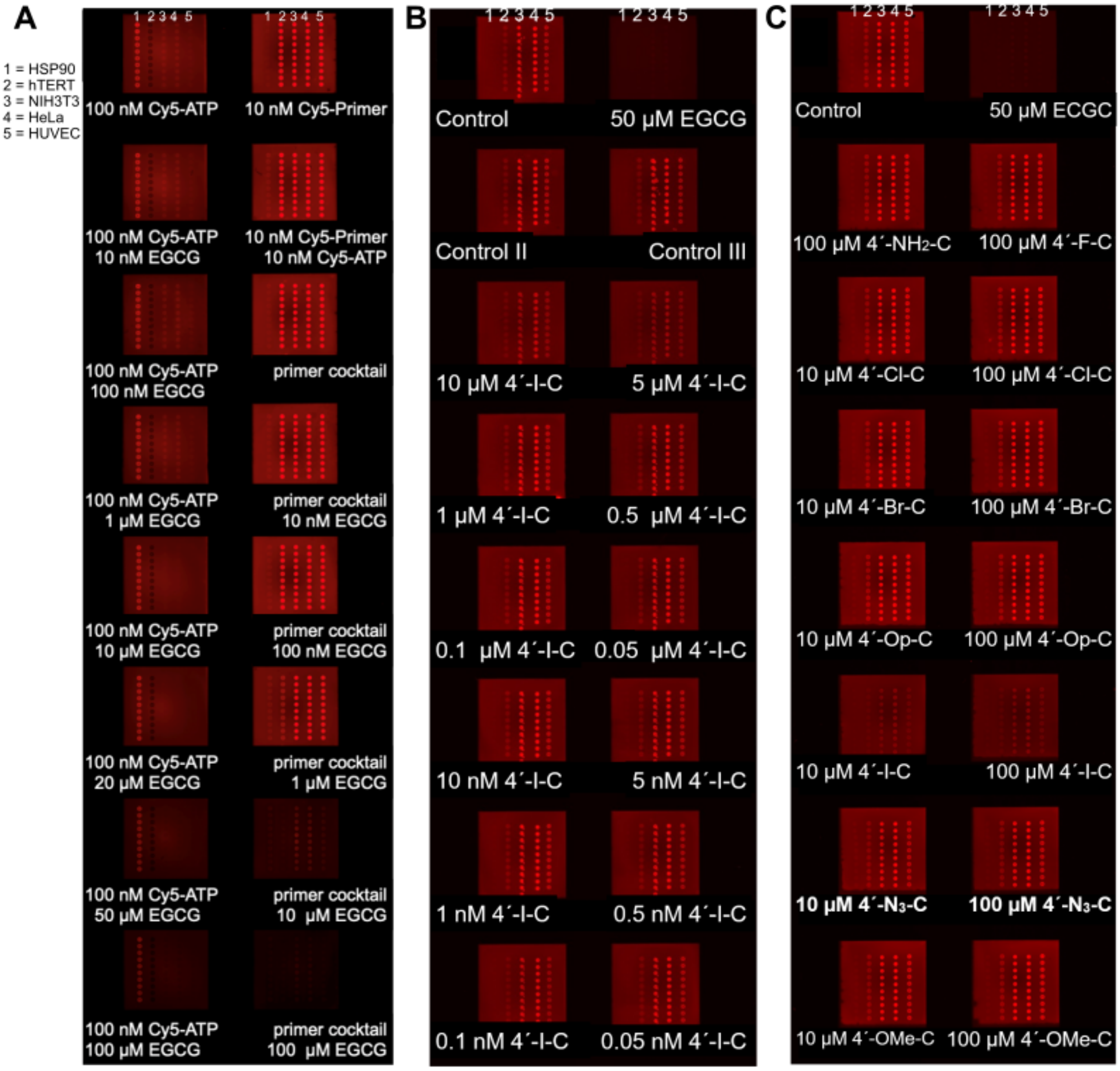

Supplement Figure 6

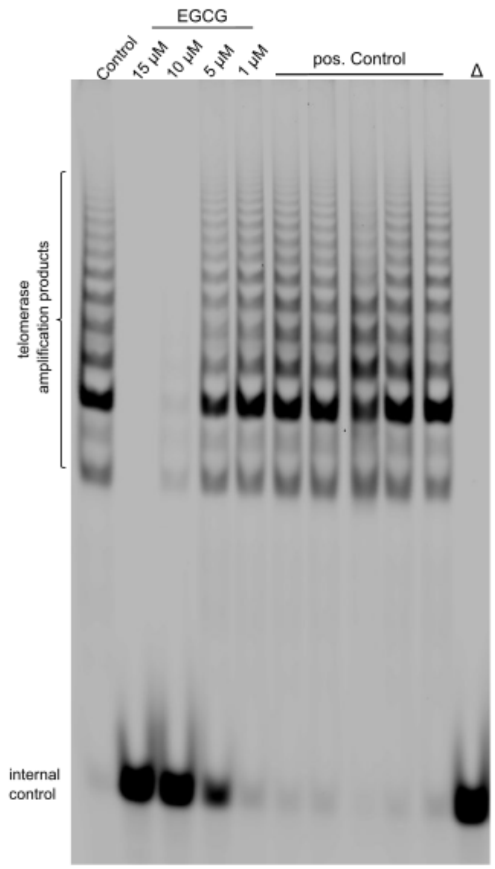

Supplement Figure 7

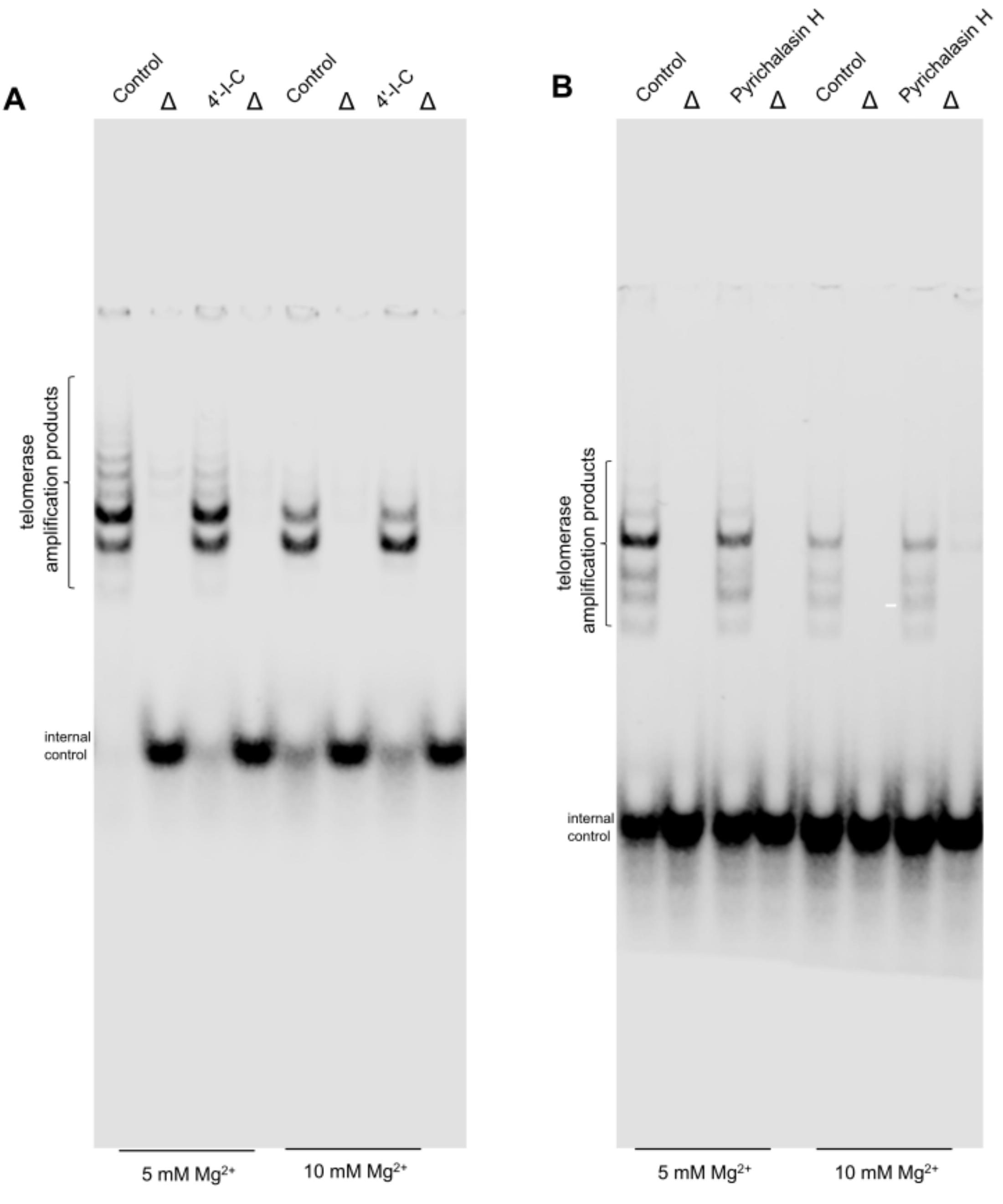

Supplement Figure 8

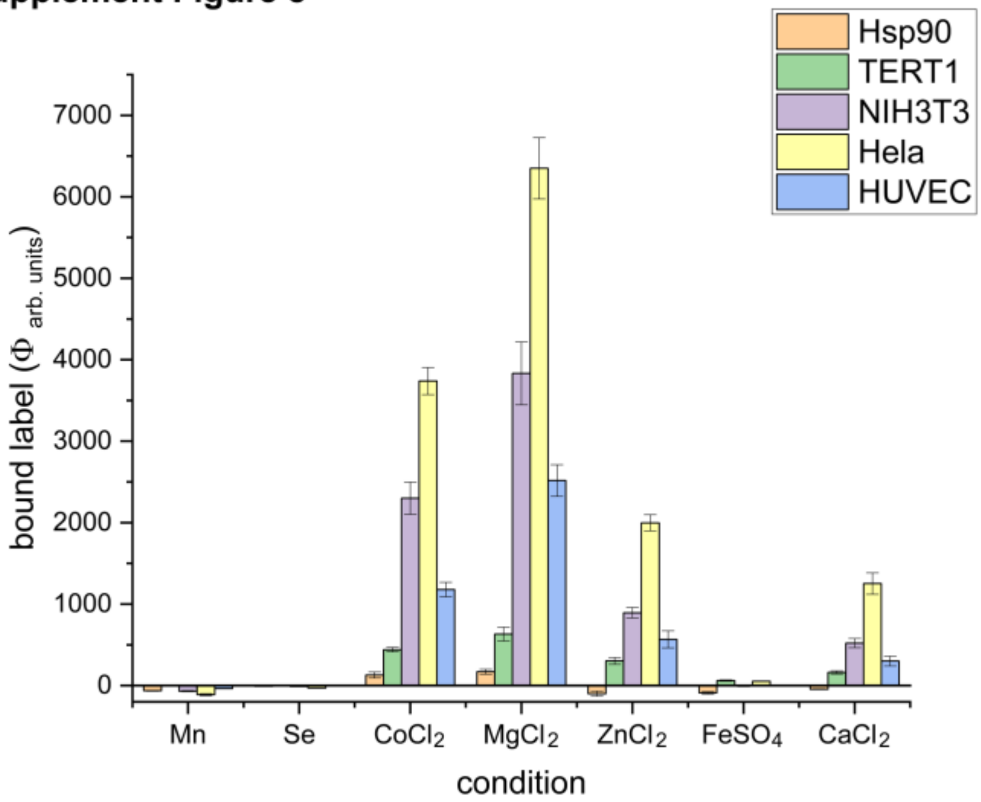

Supplement Figure 9

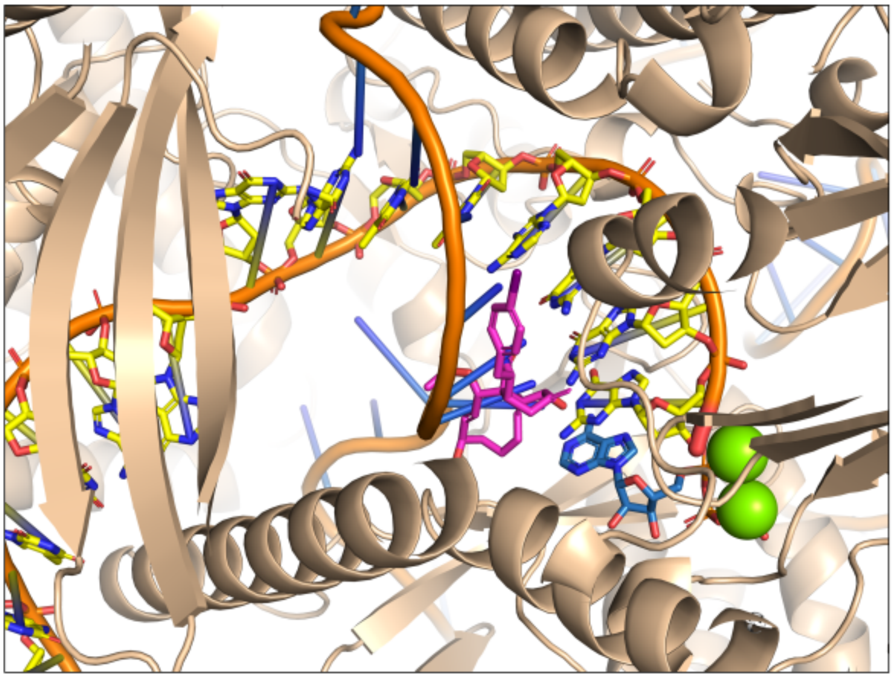

Supplement Figure 10

Chemical Characterisation of 4'-iodocytochalasin H

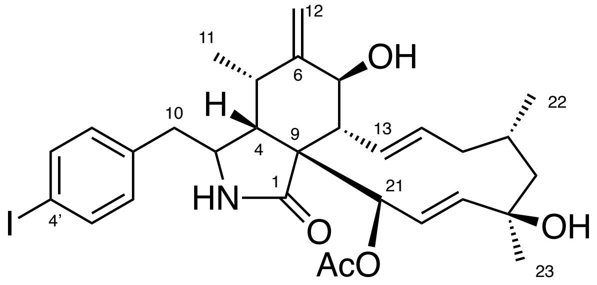

Chemical Formula: $C_{30}H_{38}INO_5$
Exact Mass: 619.1795

Position	δ_H	M	$J_{H\text{-}H}$/Hz	δ_C	HSQC	HMBC H to C	H-H COSY
1	-	-	-	174.3	-	-	-
2	5.46	brs	-	-	-	-	-
3	3.23	ddd	1.1, 4.8, 9.6	53.5	CH	1‘, 1, 4, 5	4, 5, 10
4	2.13	dd	3.6, 5.1	50.5	CH	1, 3, 5	3, 5, 10
5	2.80	m	-	32.7	CH	1’, 3	4, 11, 12
6	-	-	-	147.9	-	-	-
7	3.84	dd	1.3, 10.8	69.7	CH	5, 6, 8, 12, 13	8, 12
8	2.95	dd	9.8, 10.8	47.2	CH	7, 9, 13, 20, 21	7, 13
9	-	-	-	51.7	-	-	-
10a	2.63	dd	9.5, 13.5	45.2	CH_2	1’, 3	3, 4, 12
10b	2.82	dd	4.8, 13.5	-	-	-	-
11	0.99	d	6.7	14.0	CH_3	4, 5, 6	5
12a	5.12	brs	-	114.3	CH_2	5, 6, 7	5, 7, 11
12b	5.37	brs	-	-	-	-	-
13	5.78	ddd	1.4, 9.6, 15.5	127.1	CH	7, 8, 15	8, 14, 15
14	5.43	ddd	4.8, 10.3, 15.5	138.8	CH	9, 15	13, 15
15a	1.82	m	-	42.7	CH_2	13, 14, 16, 17	13, 14,
15b	2.05	m	-	-	-	-	-
16	1.81	m	-	28.5	CH	13, 14, 17	22
17a	1.60	m	-	53.9	CH_2	18, 19	-
17b	1.89	m	-	-	-	-	-
18	-	-	-	74.4	-	-	-
19	5.55	brs	-	138.3	CH	-	20
20	5.88	dd	2.9, 16.4	126.0	CH	18, 19	19
21	5.58	brs	-	77.2	CH	8, 9, 19, 20, 23	-
22	1.07	d	6.5	26.4	CH_3	15, 16, 17	16
23	1.37	s	-	31.1	CH_3	17, 18, 19	-
1‘	-	-	-	131.0	-	-	-
2’ 6’	6.93	d	8.2	131.1	2 x CH	3’, 4’, 5’, 10	3’, 5’
3’ 5’	7.67	d	8.2	138.0	2 x CH	1’, 4’	2’, 6’
4’	-	-	-	137.3	-	-	-
24	-	-	-	170.2	-	-	-
25	2.27	s	-	20.9	CH_3	21, 24	-

Table S2: NMR data of 4´-iodocytochalasin H recorded at 400 MHz in $CDCl_3$. Values are in agreement with published data

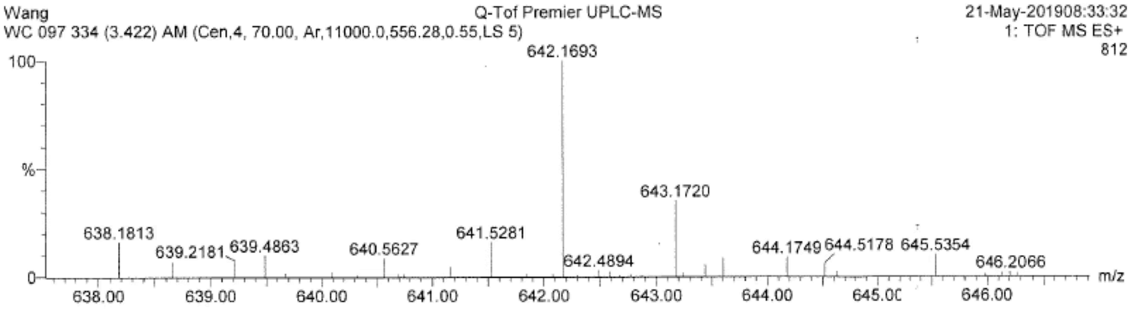

	Calc. Mass	mDa	PPM	DBE	i-FIT	Formula
Minimum:				-0.5		
Maximum:		5.0	20.0	60.0		
Mass	Calc. Mass	mDa	PPM	DBE	i-FIT	Formula
642.1693	642.1692	0.1	0.2	11.5	2.1	C30 H38 N O5 Na I
	642.1706	-1.3	-2.0	16.5	2.5	C31 H34 N5 O Na I
	642.1676	1.7	2.6	10.5	3.7	C27 H37 N3 O7 I

HRMS (*m/z*): calculated for [$C_{30}H_{38}NO_5I$ + Na]: 642.1692, found: 642.1693.

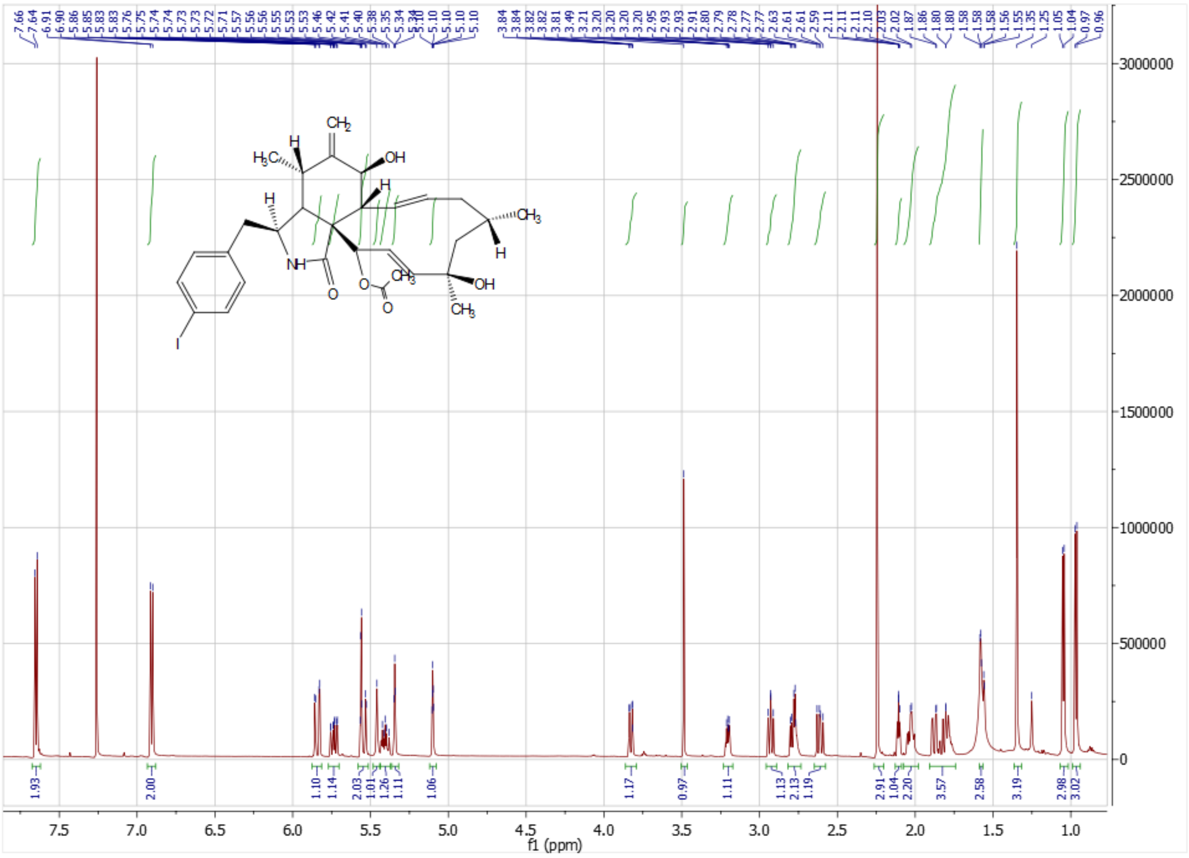

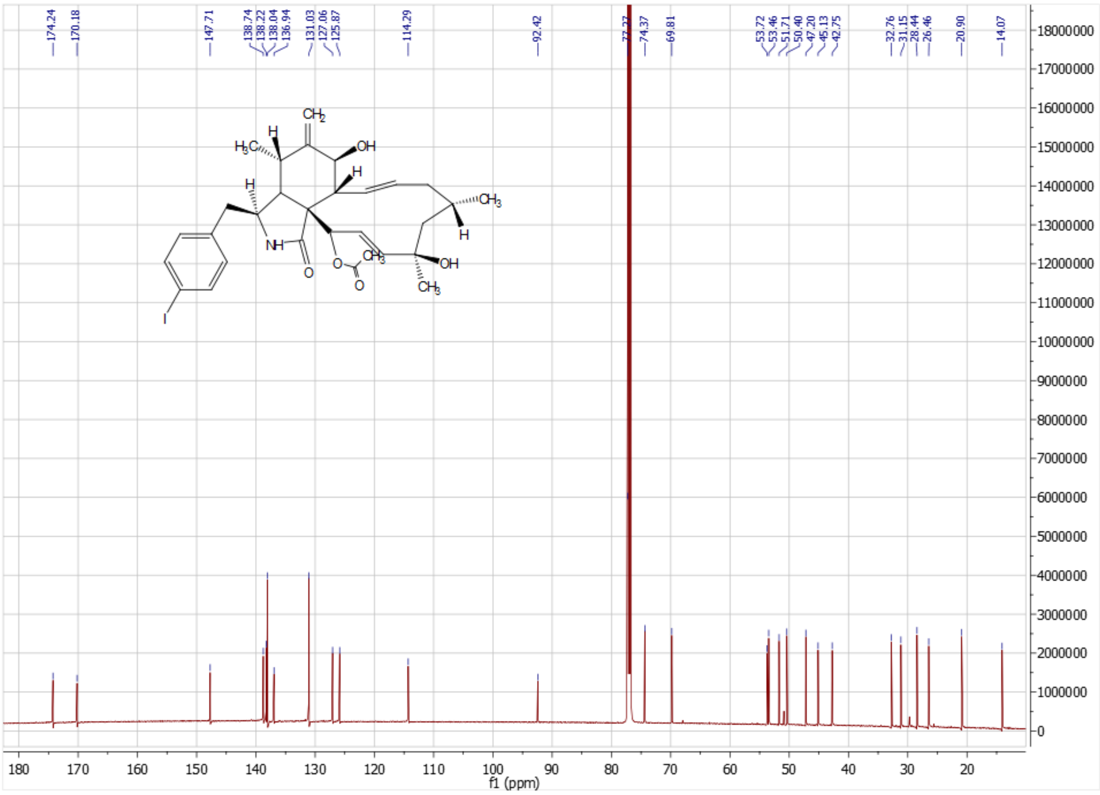
174.24
170.18
147.71
138.74
138.22
138.04
136.94
131.03
127.06
125.87
114.29
92.42
74.37
69.81
53.72
53.46
51.71
50.40
47.20
45.13
42.75
32.76
31.15
28.44
26.46
20.90
14.07
f1 (ppm)

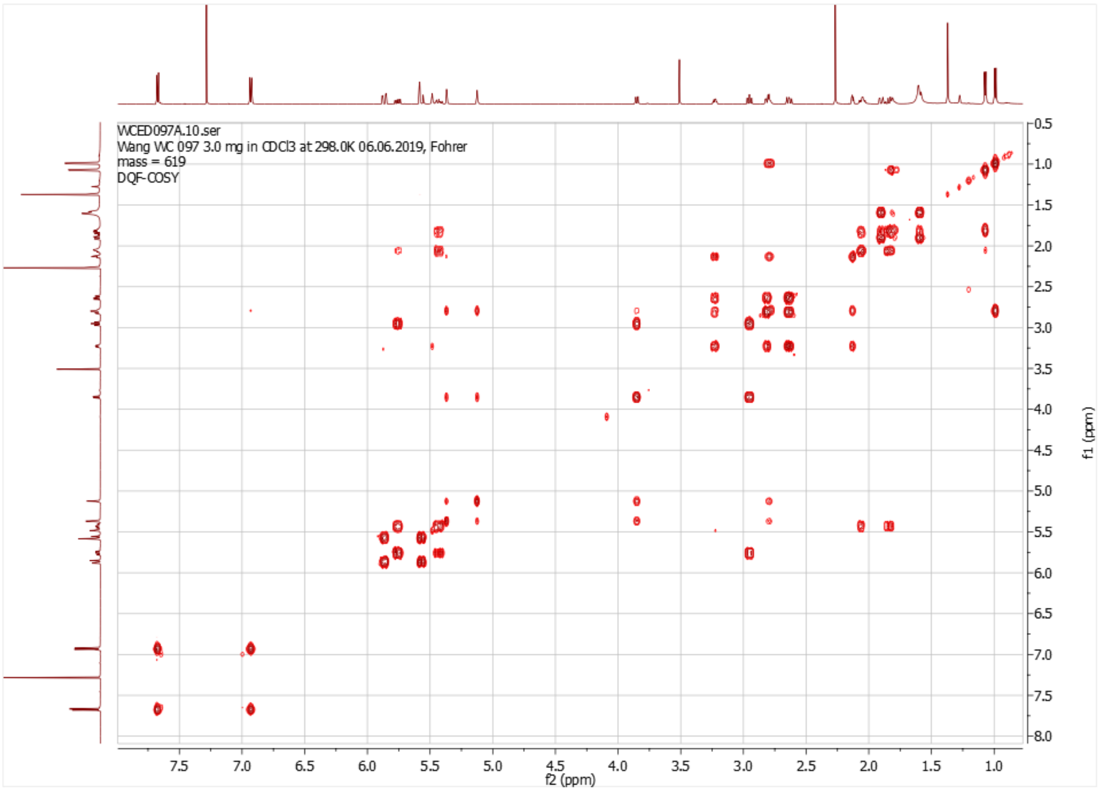
WCED097A.10.ser
Wang WC 097 3.0 mg in CDCl3 at 298.0K 06.06.2019, Fohrer
mass = 619
DQF-COSY
f2 (ppm)
f1 (ppm)

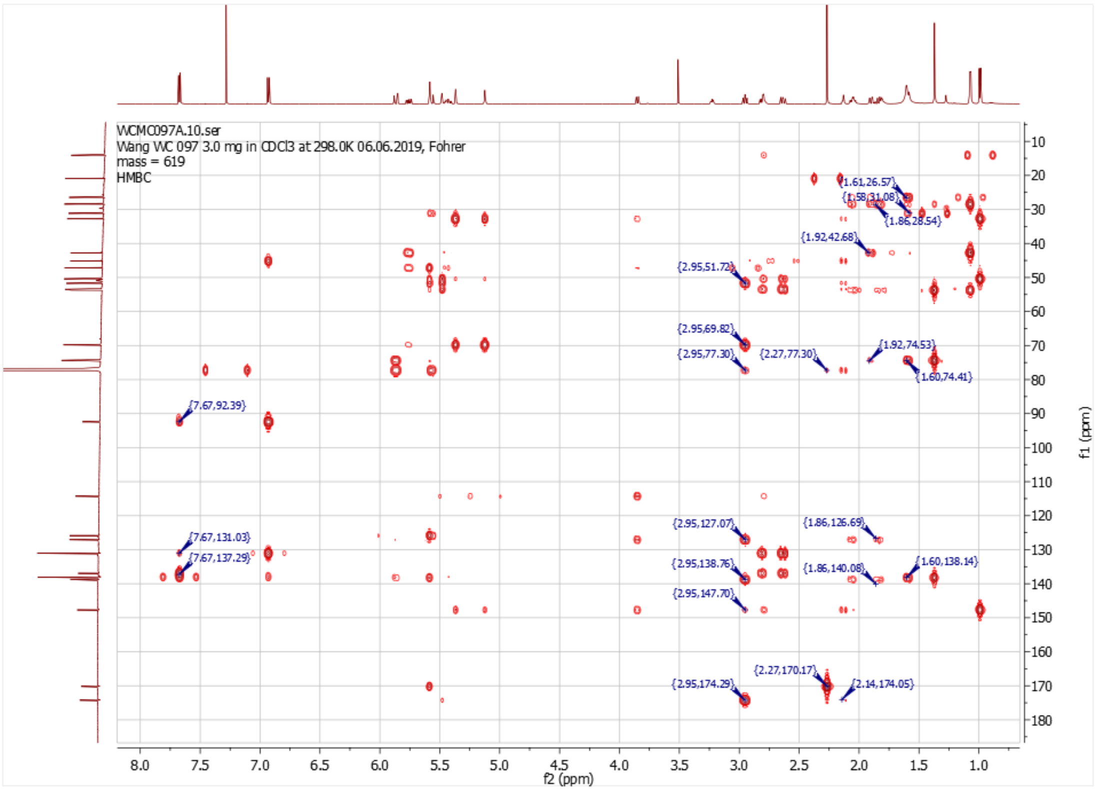
WCMC097A.10.ser
Wang WC 097 3.0 mg in CDCl3 at 298.0K 06.06.2019, Fohrer
mass = 619
HMBC
f2 (ppm)
f1 (ppm)

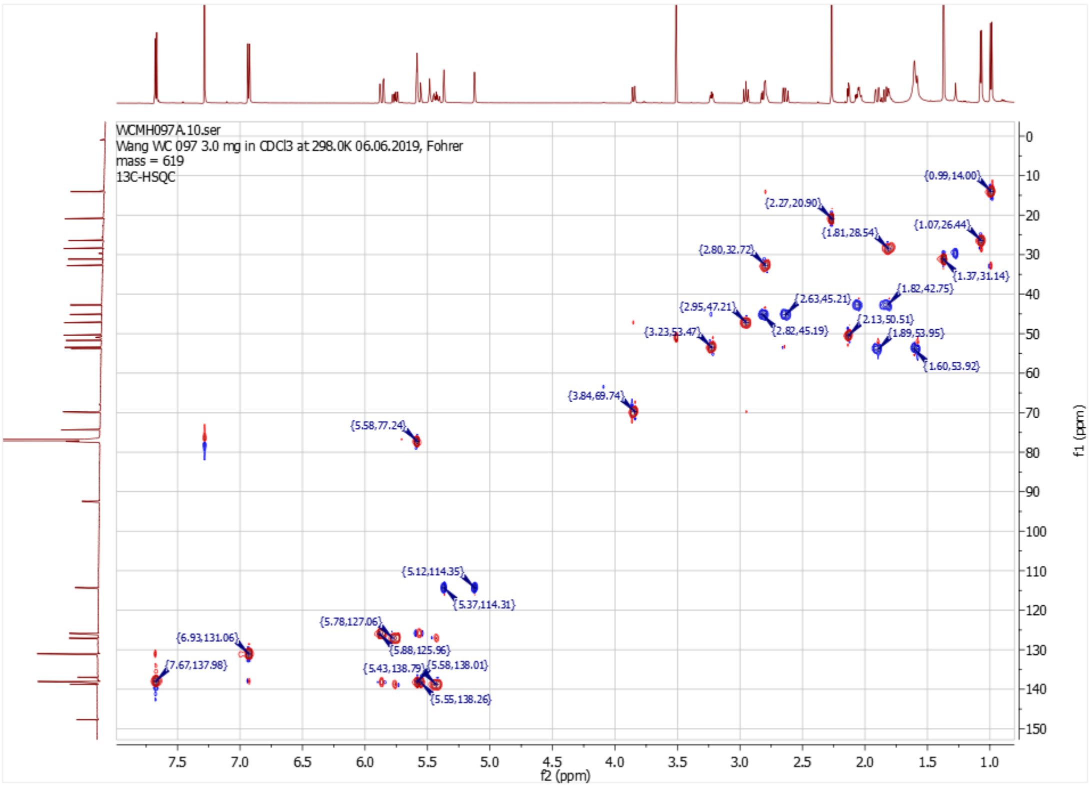
WCMH097A.10.ser
Wang WC 097 3.0 mg in CDCl3 at 298.0K 06.06.2019, Fohrer
mass = 619
13C-HSQC
f2 (ppm)
f1 (ppm)
